# NIN-like protein 8 is a master regulator of nitrate-promoted seed germination in *Arabidopsis*

**DOI:** 10.1038/ncomms13179

**Published:** 2016-10-12

**Authors:** Dawei Yan, Vanathy Easwaran, Vivian Chau, Masanori Okamoto, Matthew Ierullo, Mitsuhiro Kimura, Akira Endo, Ryoichi Yano, Asher Pasha, Yunchen Gong, Yong-Mei Bi, Nicolas Provart, David Guttman, Anne Krapp, Steven J. Rothstein, Eiji Nambara

**Affiliations:** 1 Department of Cell & Systems Biology, University of Toronto, Toronto, Ontario, Canada M5S3B2; 2 Arid Land Research Center, Tottori University, Tottori 680-0001, Japan; 3 PRESTO, Japan Science and Technology Agency, Saitama 332-0012, Japan; 4 Faculty of Life and Environmental Sciences, University of Tsukuba, Tsukuba 305-8572, Japan; 5 Centre for the Analysis of Genome Evolution and Function, University of Toronto, Toronto, Ontario, Canada M5S3B2; 6 Department of Molecular and Cellular Biology, University of Guelph, Guelph, Ontario, Canada N1G2W1; 7 Institut Jean-Pierre Bourgin, INRA, AgroParisTech, CNRS, Université Paris-Saclay, RD10, 78026 Versailles, France

## Abstract

Seeds respond to multiple different environmental stimuli that regulate germination. Nitrate stimulates germination in many plants but how it does so remains unclear. Here we show that the *Arabidopsis* NIN-like protein 8 (NLP8) is essential for nitrate-promoted seed germination. Seed germination in *nlp8* loss-of-function mutants does not respond to nitrate. *NLP8* functions even in a nitrate reductase-deficient mutant background, and the requirement for *NLP8* is conserved among *Arabidopsis* accessions. NLP8 reduces abscisic acid levels in a nitrate-dependent manner and directly binds to the promoter of *CYP707A2*, encoding an abscisic acid catabolic enzyme. Genetic analysis shows that NLP8-mediated promotion of seed germination by nitrate requires CYP707A2. Finally, we show that NLP8 localizes to nuclei and unlike NLP7, does not appear to be activated by nitrate-dependent nuclear retention of NLP7, suggesting that seeds have a unique mechanism for nitrate signalling.

## Abstract (web summary)

Nitrate stimulates seed germination in many plant species. Here, Yan *et al*. show that the *Arabidopsis* transcription factor NIN-like protein 8 is required to stimulate germination in response to nitrate and induces expression of an enzyme involved in ABA catabolism.

## Graphical abstract

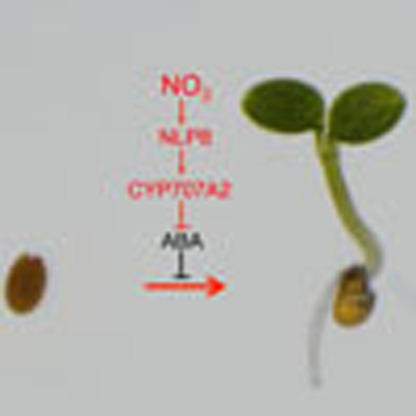

Seeds sense and respond to environmental cues such as nitrate, light, after-ripening and temperature and these determine whether the environmental conditions are suitable for germination. A transcriptome analysis revealed that different germination stimuli trigger a similar transcriptome pattern^1^. This suggests that these environmental factors regulate a common downstream event, such as plant hormone action. Abscisic acid (ABA) and gibberellins (GA) regulate seed germination antagonistically in many plant species including *Arabidopsis*. Consistently, ABA and GA metabolism and signalling respond to changing environmental factors and induce downstream events suitable for the given environment. Seed germination is negatively regulated by ABA, which accumulates at high levels in the dry seed, and thus must be degraded for germination to occur^2^. The ABA 8′-hydroxylase, CYP707A, plays a key role in ABA catabolism in various plant responses^3^. In *Arabidopsis*, CYP707A1 and CYP707A2 have distinct roles in regulating seed dormancy and germination. CYP707A1 plays a role in the degradation of ABA that occurs during the mid-maturation stage of seed development. In contrast, *CYP707A2* is expressed during the late-maturation stage of seed development, and becomes highly expressed after seed imbibition occurs^4^. Mutants of *cyp707a1* over-accumulate ABA in the dry seed, while those of *cyp707a2* accumulate only slightly higher levels of ABA in the dry seed, and show a defect in the ability to reduce ABA content once seed imbibition has occurred. Both mutants maintain a higher ABA content for a more prolonged period of time during seed imbibition when compared with the wild type, and are thus hyper-dormant. The expression of *CYP707A2* is controlled by germination-related signals, suggesting that CYP707A2 acts as a hub for environmental signalling in germinating seeds^5^,^6^,^7^. Despite this, not much is known about how the expression of *CYP707A2* is regulated by environmental factors.

Nitrate is the primary nitrogen source for plants and is assimilated to nitrite, ammonium and amino acids^8^. Nitrate reductase (NR) catalyses the conversion of nitrate to nitrite, the committed step of nitrate assimilation. In addition, nitrate acts as a signal molecule in that it induces a rapid shift in transcriptomes, even at low concentrations^9^. Nitrate regulates numerous aspects of plant developmental processes such as seed germination, root architecture and flowering^10^,^11^,^12^. Nitrate promotes seed germination independently of its reduction by NR, indicating it acts as a signal^10^,^11^. In addition to nitrate, other nitrogen-containing compounds such as nitrite, nitric oxide (NO) and cyanides also promote *Arabidopsis* seed germination^13^. A pharmacological experiment showed that nitrate promotion of *Arabidopsis* seed germination was blocked by 2-(4-carboxyphenyl)-4,4,5,5-tetramethylimidazoline-1-oxyl-3-oxide (cPTIO), an NO-specific scavenger^14^. On the basis of this result, it was argued that nitrate promotion of seed germination is mediated by NO signalling. However, this result assumes that nitrate acts in a linear pathway that is upstream of NO signalling, and not in parallel or distinct pathways. Recently, Gibbs *et al*. reported that to promote germination, NO action requires the N-end rule proteasome pathway^15^. Identification of the nitrate signalling components in seeds, is crucial for evaluating the mechanism of nitrate action on germination in relation to other germination stimulating signalling pathways including NO.

In *Arabidopsis*, several regulators for nitrate signalling have been identified. Chlorate-resistant1 (CHL1, NRT1.1, NPF6.3) is a dual-affinity nitrate transporter and also acts as a nitrate sensor that is able to sense a wide range of nitrate concentrations through the phosphorylation of T101 (ref. 16). Several transcription factors including *Arabidopsis* nitrate regulated 1 (ANR1), Teosinte branched1/cycloidea/proliferating cell factor1-20 (TCP20) and NIN-like protein (NLP) have been shown to be involved in nitrate responses^17^,^18^,^19^,^20^. ANR1 is a MADS-box transcription factor controlling the growth of lateral roots and is believed to act downstream of CHL1 in response to a locally enriched nitrate source^17^,^21^. In contrast, TCP20 has been implicated in systemic nitrate signalling^18^. Recently, NLPs have been shown to play a central role in nitrate-regulated gene expression, nitrate assimilation and nitrate-induced growth promotion^20^,^22^. NLPs have been shown to directly bind to the nitrate-responsive *cis*-element (NRE) to induce nitrate-mediated transcription^20^. The N-terminal region of NLP6 acts as a nitrate-dependent transcriptional activation domain^20^. The *nlp7* mutants display nitrate-starvation phenotypes when nitrate is used as the only nitrogen source^19^. Interestingly, nitrate regulates NLP7 by mediating its localization and retention in the nucleus. Primary nitrate-responsive genes such as those responsible for nitrate transport (for example, *NRT1.1*, *NRT2.1*) and assimilation (for example, *NIA1*, *NIA2*) are common direct targets of NLPs and TCP20 (refs 18, 22). However, it remains unknown how the upstream signalling influences a wide range of nitrate responses.

Here we report the identification and characterization of NLP8 in nitrate-promoted seed germination. Our research indicates that NLP8 regulates nitrate-promoted germination and directly activates expression of *CYP707A2*, an ABA catabolic enzyme. In addition, our research also suggests that NLP8 is activated by nitrate signalling by a distinct mechanism from the control of NLP7 by nitrate-dependent nuclear retention. The role and mechanism of NLP8-mediated regulation of nitrate-promoted seed germination is discussed.

## Results

### NLP8 is required for nitrate-promoted seed germination

Columbia (Col-0) wild-type seeds of *Arabidopsis* are dormant when harvested from plants grown at 16 °C (refs 23, 24). The dormant Col-0 seeds did not germinate when imbibed in water, but germinated in the presence of 1 mM KNO_3_. We utilized this system to investigate the nitrate response in seed germination. We previously reported that nitrate-induced gene expression occurs in 6-h imbibed seeds^25^. Therefore, we hypothesized that seeds imbibed for a short period of time (within 6 h), contain all components necessary for nitrate signalling. On the basis of the microarray data from seeds imbibed for <6 h (ref. 26), we selected candidate regulators for nitrate signalling in seeds and analysed whether or not corresponding T-DNA insertion mutants displayed nitrate-induced seed germination. Among the mutant lines examined, mutants defective in *NIN-like protein8* (*NLP8*; At2g43500) did not germinate in the presence of 1 mM KNO_3_.

**Figure 1:**
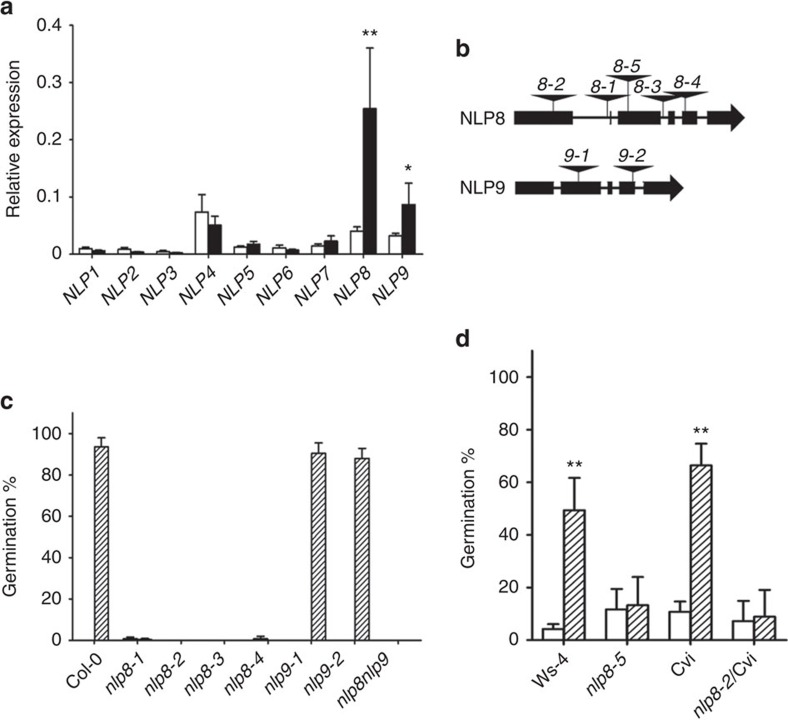
Nitrate promotes seed germination in an NLP8-dependent manner. (**a**) Relative expression level of NLPs in dry seeds (white bar) and 6-h imbibed Col-0 seeds (black bar) from 16 °C. Transcript levels were normalized to the Col-0 genomic DNA and are shown by a mean±s.d. (*n*=4). *P<0.05; **P<0.01 (Student's *t*-test compared with the relative expression in dry seeds). (**b**) Locations of T-DNA insertions in the *NLP8* and *NLP9*. (**c**) Germination of *nlp8* and *nlp9* mutants in the presence of nitrate. Wild type, *nlp8*, *nlp9*, *nlp8-2nlp9-1* mutants of Col-0 background were grown at 16 °C, harvested, and stored for 2 weeks at room temperature. Seeds were imbibed in water with 1 mM KCl (white bar) or KNO_3_ (lined bar) for 7 days. Percentage of germination is shown by a mean±s.d. (*n*=3). Note that all samples did not germinate in water with 1 mM KCl, thus the white bars are invisible. (**d**) Germination of *nlp8* mutants of Ws-4 and Cvi backgrounds in the presence of nitrate. Seeds were harvested from plants grown at 22 °C. Freshly harvested Ws-4 and *nlp8-5*, and 2-month stored Cvi and *nlp8-2*/Cvi were used for germination tests. Seeds were imbibed in water with 1 mM KCl (white bar) or KNO_3_ (lined bar) for 7 days. Percentage of germination is shown by a mean±s.d. (*n*=3). **P<0.01 (Student *t*-test compared with corresponding wild type).

The *Arabidopsis thaliana* genome encodes nine NLP family members^27^. Quantitative reverse transcription PCR (qRT–PCR) analysis showed that *NLP8* was highly induced in imbibed seeds and the most abundantly expressed *NLP*s in 6-h imbibed seeds (Fig. 1a). Expression analysis also showed that nitrate does not regulate the mRNA level of NLP8 during seed germination (Supplementary Fig. 1). The *nlp8* (*nlp8-1* to *nlp8-4)* and *nlp9* (*nlp9-1* and *nlp9-2)* seeds of Col-0 background grown at 16 °C were used for germination tests (Fig. 1b). Col-0 and *nlp9* mutants showed nitrate-promoted germination, however four *nlp8* alleles did not (Fig. 1c). The *nlp8-2nlp9-1* double mutant showed no germination in the presence of KNO_3_ (Fig. 1c). These results indicate that NLP8 is required for nitrate-promoted seed germination.

We then investigated whether the role of NLP8 was conserved across accessions. Wassilewskija-4 (Ws-4) and Cape Verde Islands (Cvi) accessions produce dormant seeds even harvested from plants grown at 22 °C. Seeds of Ws-4 wild-type and *nlp8-5* mutant in the Ws-4 background harvested from plants grown at 22 °C were tested to determine whether germination could be promoted by nitrate (Fig. 1b). Ws-4 seeds, but not *nlp8-5* seeds, responded to nitrate (Fig. 1d). We then tested the effect of the *nlp8* mutation in the Cvi background. *nlp8-2* was crossed to Cvi and near-isogenic lines were isolated after four backcross generations for the *nlp8-2* mutation in the Cvi background (*nlp8-2*/Cvi) (Supplementary Fig. 2). Germination tests were performed using *nlp8-2*/Cvi and Cvi seeds. We found that the *nlp8-2* mutation reduced the seed nitrate response in the Cvi background (Fig. 1d), showing that the requirement for NLP8 in nitrate-promoted germination is conserved across *Arabidopsis* accessions.

### NLP8 is involved in nitrate signalling during germination

To examine the specificity of the *nlp8* mutant phenotype to nitrate, the effect of stratification was tested. Stratified *nlp8-1*, *nlp8-2* and *nlp8-2nlp9-1* were able to germinate without application of KNO_3_, which was similar to that of the wild type (Supplementary Fig. 3). This indicates that *nlp8* mutants are able to respond to the stratification treatment.

**Figure 2:**
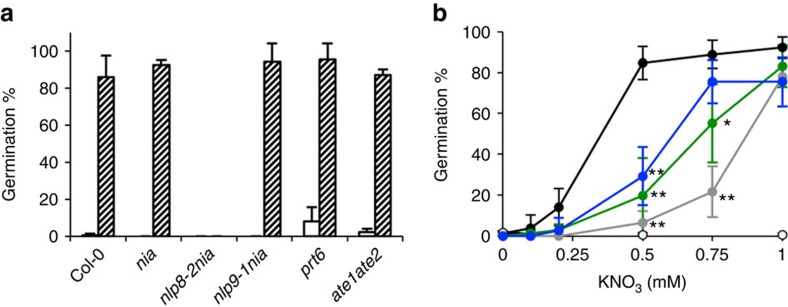
NLP8 plays a role in nitrate-specific signalling. (**a**) Germination of *nia1nia2* nitrate reductase mutant (*nia*), *nlp8-2nia1nia2* (*nlp8-2nia*) and *nlp9-1nia1nia2* (*nlp9-1nia*) triple mutants, NO-insensitive *ate1ate2* and *prt6* mutants with or without nitrate. Percentage of germination is shown by a mean±s.d. (*n*=3). A white and lined bar indicates percentage of germination in water with 1 mM KCl and KNO_3_, respectively. (**b**) Nitrate dose responses of nitrate transceptor mutant lines, *chl1-5*, *T101A/chl1-5* and *T101D/chl1-5*, and the *nlp8-2* mutant during seed germination. Percentage of germination is shown by a mean±s.d. (*n*=3). Wild type, black; *chl1-5*, gray; *T101A/chl1-5*, green; *T101D/chl1-5*, blue; *nlp8-2*, white. *P<0.05; **P<0.01 (Student *t*-test compared with Col-0 wild type).

NR mutants are defective in both nitrate assimilation and nitric oxide (NO) production^28^,^29^. To distinguish if the effect of NLP8 on nitrate-regulated germination is triggered by direct nitrate signalling or nitrate assimilation and its products, *nlp8-2nia1nia2* and *nlp9-1nia1nia2* triple mutants were made. Germination of the *nia1nia2* and *nlp9-1nia1nia2* mutants was promoted upon application of nitrate (Fig. 2a). However, the *nlp8-2nia1nia2* triple mutant did not respond to nitrate (Fig. 2a). This indicates that the role of NLP8 in nitrate-promoted germination involves nitrate signalling, rather than nitrate assimilation or NO production. NO-induced seed germination was shown to be mediated by the N-end rule proteasome degradation pathway, and two enzymes, Proteolysis 6 (PRT6) and Arg-tRNA protein transferase (ATE), were recently shown to be required for NO-promoted seed germination^15^. The *prt6* and *ate1ate2* mutants were still sensitive to nitrate-promoted seed germination (Fig. 2a), suggesting that the nitrate and NO signalling pathways are distinct from one another in seed germination.

CHL1 was characterized as a nitrate sensor in *Arabidopsis*, and phosphorylation at T101 is important for nitrate transport and response^16^. The *chl1* mutant was shown to be less sensitive to nitrate during seed germination^10^. Therefore, we compared the nitrate responsiveness between *nlp8* and *chl1* mutants during germination. The *chl1-5* mutant contains a deletion that eliminates *CHL1*, while *T101A/chl1-5* and *T101D/chl1-5* lines contain the *CHL1* mutant genes with T101A and T101D, respectively, in the *chl1-5* mutant background^16^. *chl1-5*, *T101A/chl1-5* and *T101D/chl1-5* were tested for their nitrate response in seed germination. Similar to the previous report^10^, *chl1-5* is less sensitive to nitrate-promoted seed germination compared with the Col-0 control (Fig. 2b). However, the insensitivity of *chl1-5* to nitrate was only observed at low concentrations of nitrate, while *nlp8-2* displayed a more prominent insensitivity to nitrate (Fig. 2b). This indicates that NLP8 regulates a wider range of nitrate responses than CHL1 does during seed germination. The negligible difference in germination phenotypes between *chl1-5* and *T101A/chl1-5* or *T101D/chl1-5* suggested that the phosphorylation status of T101 of CHL1 has little effect on nitrate-promoted seed germination (Fig. 2b).

### NLP8 regulates gene expression in response to nitrate

**Figure 3:**
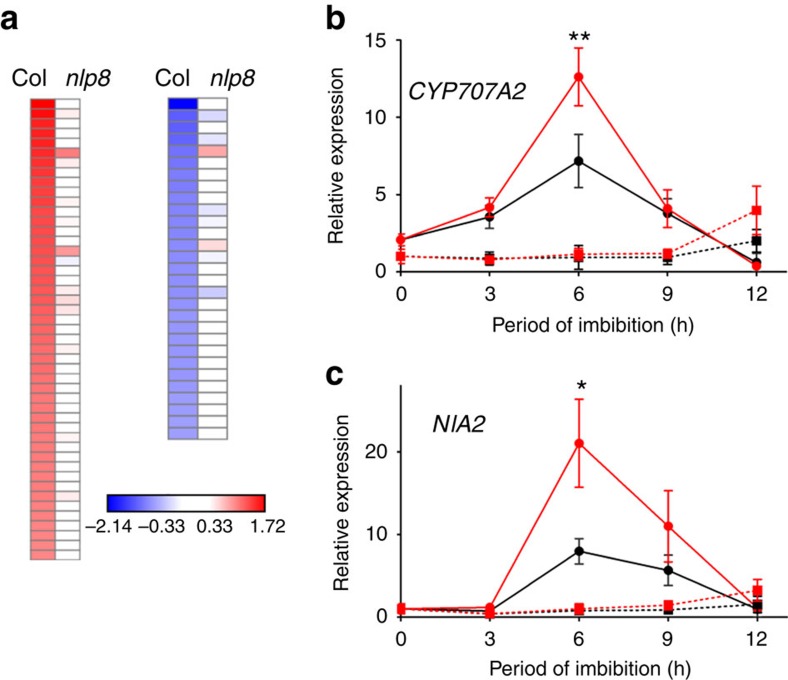
NLP8 is a master regulator for nitrate-induced gene expression during seed germination. (**a**) Nitrate-upregulated (left) and downregulated (right) genes in 6-h imbibed seeds in Col-0 or the *nlp8-2* mutant. Seeds were imbibed in water with 1 mM KCl or KNO_3_ for 6 h and RNA was extracted for RNA-seq. The readouts from KNO_3_-treated seeds were normalized to those from KCl control and a mean of two biological repeats was used to generate a heatmap. (**b**,**c**) qRT–PCR expression analysis of *CYP707A2* (**b**) and of *NIA2* (**c**) in Col-0 and *nlp8-2* seeds. The transcript levels were normalized to the expression level of At1g13320 and are shown by a mean±s.d. (*n*=3). Col-0 imbibed in KCl, black circle with solid line; Col-0 imbibed in KNO_3_, red circle with solid line; *nlp8-2* imbibed in KCl, black square with dotted line; *nlp8-2* imbibed in KNO_3_, red square with dotted line. *P<0.05, **P <0.01; Student's *t*-test compared with the relative expression without nitrate in the same genotype.

An RNA-seq experiment was performed using RNA extracted from Col-0 and *nlp8-2* seeds imbibed for 6 h with 1 mM KNO_3_ or KCl (Supplementary Data 1). Forty seven upregulated genes and twenty-nine downregulated genes were identified in 6-h imbibed Col-0 seeds (Fig. 3a and Supplementary Fig. 4). Gene ontology (GO) term distribution indicates genes encoding transcription factors (eight genes, *P*=0.038) and related to nitrogen metabolism (six genes, *P*=6.47e-6) are overrepresented among those genes upregulated by nitrate. Some of the known nitrate-inducible genes such as *NIA1*, *NIA2*, *nitrite reductase1* (*NIR1)*, root-type *Ferredoxin:NADP(H) Oxidoreductase1* (RFNR1), *RFNR2*, *Glucose-6-phosphate dehydrogenase2* (*G6PD2*) and G2-like transcription factor (AT1G25550) were included in the upregulated genes. In addition as we previously reported, *CYP707A2*, the main ABA catabolic gene during seed germination, was induced by nitrate. No other genes related to ABA and GA metabolism and signalling were found among the list of nitrate-regulated genes (Supplementary Fig. 5). Nitrate-mediated upregulation of almost all of the nitrate-inducible genes identified by RNA-seq, was absent or was significantly decreased in the *nlp8-2* mutant (Fig. 3a). This was also true for the nitrate downregulated genes. Expression of some genes was further analysed by qRT–PCR to examine their expression kinetics on imbibition in the seed. When Col-0 seeds were imbibed in water with KCl, the expression of *CYP707A2* increased and then decreased, with its maximum expression at 6 h (Fig. 3b). The expression of *CYP707A2* in imbibed Col-0 seeds was induced by KNO_3_ with similar kinetics but at a higher level to that observed for the KCl-treated seeds. *CYP707A2* had a much lower level of expression of in *nlp8-2* in both KCl and KNO_3_ imbibed seeds. Importantly, nitrate had no effect on the expression of *CYP707A2* in *nlp8-2* (Fig. 3b). This was also found to be true for the expression of *NIA2* (Fig. 3c). In addition, the expression of another eight genes were analysed by qRT–PCR and we observed expression patterns consistent with the RNA-seq data (Supplementary Figs 6 and 7).

### NLP8 regulates ABA catabolism and induces *CYP707A2*

**Figure 4:**
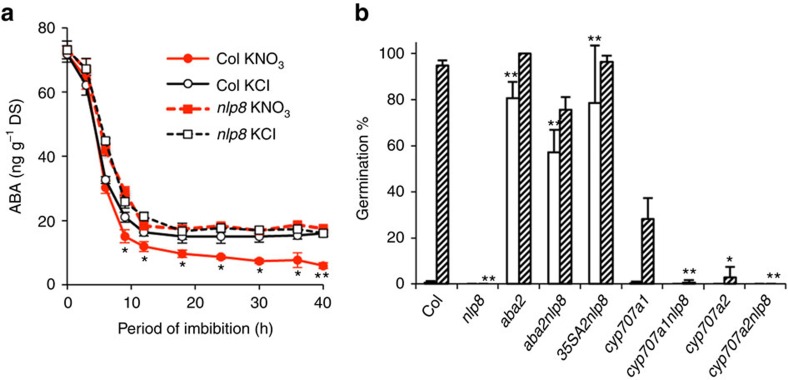
NLP8 regulates ABA catabolism during seed germination. (**a**) Quantification of ABA contents in Col-0 and *nlp8-2* seeds. Seeds were imbibed in water with 1 mM KCl or KNO_3_ for the indicated time periods. The ABA content was measured by liquid chromatography equipped with a mass spectrometry. Measurements were performed using three biological replicates, and a mean is shown with s.d. (*n*=3). Bold and dotted lines indicate Col-0 and *nlp8-2*, and black and red lines indicate KCl- and KNO_3_-treated seeds. * and ** indicate the significant differences with *P*<0.05 and *P*<0.01 (Student's *t*-test compared with the ABA level in KCl-treated samples of the same genotype), respectively. (**b**) Germination of ABA metabolism and *nlp8* mutants in the presence of nitrate. Col-0, *aba2*, *cyp707a1*, *cyp707a2* and double mutants with *nlp8-2* and *35S::CYP707A2* expressed in *nlp8-2* (*35SA2nlp8*) were imbibed in water without (white bars) or with 1 mM nitrate (lined bars). Percentage of germination is shown by a mean±s.d. (*n*=3). * and ** indicate the significant differences from that of corresponding wild type, with *P*<0.05 and *P*<0.01 (Student's *t*-test), respectively.

To determine whether the nitrate-induced decline in ABA contents is NLP8-dependent, the ABA content was measured in dry seeds and imbibed seeds of Col-0 and *nlp8-2* with 1 mM KCl or KNO_3_ (Fig. 4a). In Col-0 seeds, the nitrate-induced ABA decrease was observed at 9 h after the onset of imbibition, and the ABA content in KNO_3_-treated seeds was lower than those in KCl-treated seeds thereafter (Fig. 4a). On the other hand, the ABA content in KNO_3_-treated *nlp8-2* seeds was comparable to that seen in the KCl-treated *nlp8-2* seeds, which was equivalent to that in KCl-treated Col-0 seeds (Fig. 4a). This indicates that the ABA decrease by nitrate is NLP8-dependent. The *aba2* mutant line, which has a defect in ABA biosynthesis, was non-dormant even when harvested from 16 °C, and was able to germinate in the absence of nitrate (Fig. 4b). *aba2* was found to be epistatic to *nlp8-2* since *aba2nlp8-2* germinated even without nitrate, showing that *de novo* ABA biosynthesis is required for *nlp8-2* to establish seed dormancy. Col-0 and *cyp707a1* responded to nitrate, while *cyp707a2* was less sensitive to nitrate (Fig. 4b). Nitrate-promoted germination was not observed in the *cyp707a1nlp8-2* and *cyp707a2nlp8-2* double mutants (Fig. 4b). It is noteworthy that nitrate-independent expression of *CYP707A2* in *nlp8-2* by introducing the *35S::CYP707A2* caused nitrate-independent germination (Fig. 4b). Taken together, these results suggest that NLP8-mediated induction of *CYP707A2* promotes seed germination in response to nitrate.

**Figure 5:**
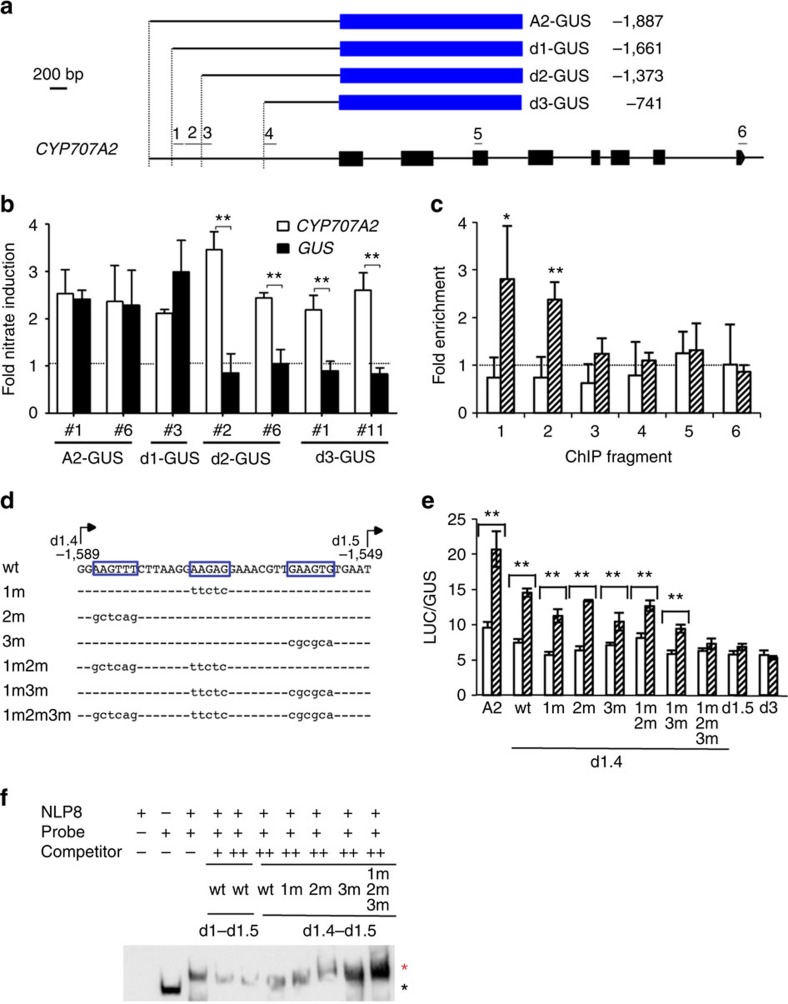
NLP8 directly binds to the *CYP707A2* promoter and triggers nitrate-induced gene expression. (**a**) The *CYP707A2* promoter-GUS constructs and the fragments used for ChIP analysis (1 to 6). (**b**) Promoter deletion analysis of *CYP707A2* for identifying promoter regions responsible for nitrate induction. qRT–PCR analysis of GUS reporter expression in 6-h imbibed seeds of promoter-GUS transgenic lines. The *GUS* transcript level in 10 mM KNO_3_-treated seeds was normalized to that in 10 mM KCl-treated seeds (black bar). The dotted line indicates the value 1 (that is, no nitrate induction). Nitrate induction of native *CYP707A2* was used as a positive control (white bar). Data shown are means±s.e. (*n*≥3). **P<0.01 (Student's *t*-test). (**c**) ChIP analysis of the *CYP707A2* promoter using *35S::NLP8-GFP* line. White bars and lined bars indicate the ChIP data from KCl-treated and KNO_3_-treated plants, respectively. Signals obtained from the anti-GFP sample (+Ab) were normalized to those from the no antibody control (−Ab). The dotted line indicates the value 1 (that is, no enrichment). Data shown are means±s.d. (*n*=4). *P<0.05; **P<0.01 (Student's *t*-test compared with corresponding KCl-treated samples). (**d**) A 41-bp region in the *CYP707A2* promoter required for nitrate induction, and three mutations introduced. (**e**) NLP8-dependent activation of the reporter (LUC) gene expression driven by mutant promoters in a protoplast system. NLP8-GFP and ACT2 (control) were transiently expressed in protoplasts in the presence of nitrate. LUC activities were normalized by GUS activities from co-introduced 35S::GUS. Averages of reporter activities of NLP8-GFP expressed protoplasts (lined bar) and ACT2 expressed protoplasts (white bar) with s.d. are shown (*n*=3). **P<0.01 (Student's *t*-test). (**f**) Specific binding of the RWP-RK domain of NLP8 to the NRE of the *CYP707A2* promoter. A biotin-labelled probe containing two copies of d1-d1.5 (1.5 ng) and RWP-RK domain of NLP8 (200 ng) were used for EMSA reactions and separated in a 5% native PAGE gel. Competitors were two copies of d1-d1.5 or d1.4-d1.5 fragments without biotin labeling (+: 50-fold, ++: 100-fold). A red star indicates the shifted band, while the black star indicates the signal from free probe.

To test whether *CYP707A2* is a direct target of NLP8, a 1.9-kb 5′ non-coding region of *CYP707A2* was cloned, fused to a GUS reporter gene (*A2-GUS*), and transformed into Col-0 (Fig. 5a). The *A2-GUS* seeds from 16 °C grown plants were tested for nitrate-induced reporter expression by qRT–PCR. The expression of the native *CYP707A2* served as a positive control for measuring nitrate-induction of the reporter gene expression. *A2-GUS* lines (#1 and #6) displayed nitrate-induced reporter expression similar to the native *CYP707A2* (Fig. 5b). Next, a promoter deletion series were made and fused to the GUS reporter gene to identify the promoter region responsible for nitrate induction (Fig. 5a). These promoter deletion constructs, *d1-GUS* to *d3-GUS*, were transformed into Col-0 and selected transgenic lines were grown at 16 °C. The *d1-GUS* (−1,661) line showed nitrate-induced reporter expression that was similar to the *A2-GUS* lines. In contrast, the *d2-GUS* (−1,373) and *d3-GUS* (−741) lines did not show nitrate-induced reporter expression (Fig. 5b). This indicates that the region between −1,661 and −1,373 is responsible for the nitrate-mediated induction of *CYP707A2*.

We next generated *35S::NLP8-GFP* transgenic lines. Six DNA fragments spanning *CYP707A2* as shown in Fig. 5a, were examined by chromatin immunoprecipitation (ChIP). The co-immunoprecipitated DNA was analysed by quantitative PCR. Relative fold enrichment was calculated by normalizing the value of immunoprecipitated anti-GFP fragments to that of the control sample that did not contain any antibody. Fragments #1 (−1,661 to −1,552) and #2 (−1,549 to −1,383) showed enrichment, but not fragments #3 to #6, in nitrate-treated samples (Fig. 5c), demonstrating a direct binding of NLP8 to these fragments in the promoter region of *CYP707A2*. Such enrichments were not observed in the KCl-treated samples (Fig. 5c). These results collectively suggest that NLP8 directly binds to the promoter of *CYP707A2* in a nitrate-dependent manner and upregulates its expression during seed germination in the presence of nitrate.

A protoplast assay was employed to further narrow down the promoter region responsible for NLP8-dependent expression of *CYP707A2*. A series of promoter deletions fused to a LUC reporter gene were tested for their nitrate induction when co-transformed with the 35S::NLP8-GFP construct. Two positive controls, A2-LUC and d1-LUC(−1,661), showed the NLP8-dependent nitrate response in the protoplast assay similar to that observed in the *A2-GUS* and *d1-GUS* stable transgenic lines (Fig. 5b and Supplementary Fig. 8). Nitrate-induced reporter expression was also observed in d1.2-LUC(−1,638), d1.3-LUC(−1,615) and d1.4-LUC(−1,589) but not in d1.5-LUC(−1,549) (Supplementary Fig. 8), indicating that the 41 bp region between d1.4 and d1.5 is responsible for NLP8-dependent nitrate induction (Fig. 5d).

To identify the NLP8-binding sites, mutations were introduced to d1.4-LUC and the NLP8-dependent reporter expression was analysed in the presence of nitrate in the protoplast assay (Fig. 5d,e). d1.4-LUC with disruptions of single sites (1m, 2m, 3m) and double sites (1m2m, 1m3m) still responded to NLP8, but the d1.4-LUC with all three sites mutated (1m2m3m) did not respond (Fig. 5e). This result suggests that these three sites are required for NLP8-dependent gene expression.

We next performed an electrophoretic mobility shift assay (EMSA) to verify the physical interaction between NLP8 and the promoter of *CYP707A2*. The binding of the RWP-RK domain of NLP8 to the d1-d1.5 fragment resulted a shifted band in the EMSA (Supplementary Fig. 9a). The application of nitrate did not affect its binding (Supplementary Fig. 9a). The shifted band disappeared by addition of competitor d1.4-d1.5 (Fig. 5f and Supplementary Fig. 9a), but mutant competitor with all three sites disrupted failed to compete (Fig. 5f and Supplementary Fig. 9b). These collectively indicate that NLP8 binds to these three sites in the *CYP707A2* promoter.

### Nitrate activates NLP8 and NLP7 via distinct mechanisms

**Figure 6:**
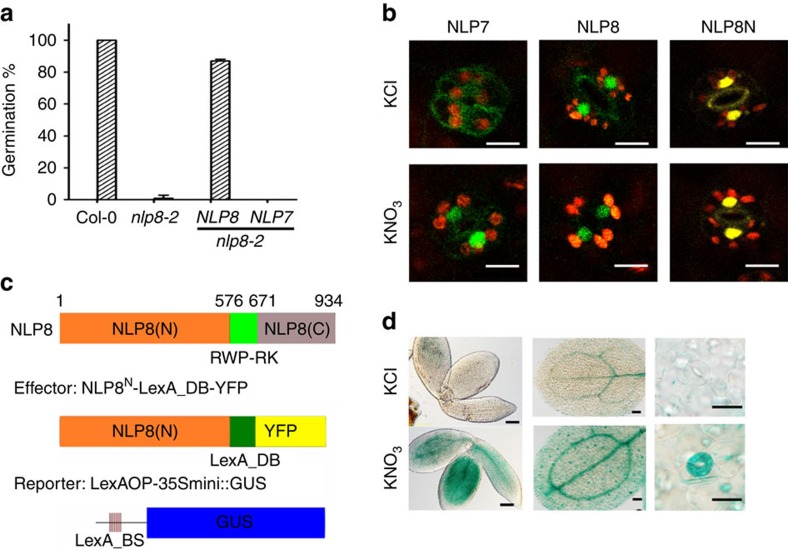
Nitrate regulates NLP8 post-transcriptionally through its N-terminal region. (**a**) Complementation of *nlp8-2* by *35S::NLP8-GFP*, but not by *35S::NLP7-GFP*. Percentage of germination is shown by a mean±s.d. (*n*=3). (**b**) Subcellular localization of NLP8-GFP and NLP8N-LexA_DB-YFP in the stomata of KCl- or KNO_3_-treated cotyledons. NLP7-GFP was used as a control for the nitrate-regulated nuclear retention. A bar indicates 10 μm. (**c**) Schematic diagram of effector construct (NLP8N-LexA_DB-YFP) harboring the N-terminal region of NLP8 (NLP8(N)), LexA DNA-binding domain (LexA_DB) and YFP, while the reporter is GUS driven by eight copies of LexA operon fused to 35S minimal promoter (LexAOP-35Smini::GUS). (**d**) GUS staining of transgenic lines harbouring both effector (NLP8N-LexA_DB-YFP) and reporter (LexAOP-35Smini::GUS). Left panel, 10 mM KCl- and KNO_3_-treated 18-h-imbibed embryos; middle panel, cotyledons of 7-day-old seedlings treated with 3 mM KCl and KNO_3_; guard cells at the cotyledons of 7-day-old seedlings treated with 3 mM KCl and KNO_3_. From left to right, bars indicate 100, 100 and 20 μm.

A *35S::NLP8-GFP* construct was introduced into *nlp8-2* by crossing them together, and the homozygous lines for both *35S::NLP8-GFP* and the *nlp8-2* mutation were selected for and designated as *NLP8-GFP nlp8-2*. *NLP8-GFP nlp8-2* was able to germinate in the presence of nitrate (Fig. 6a), showing that *NLP8-GFP* complemented *nlp8-2*. It was also found that the constitutive expression of *NLP8-GFP* did not confer nitrate-independent germination, but that germination of *NLP8-GFP nlp8-2* still required nitrate. *NLP8-GFP* also failed to alleviate the weak reduced nitrate sensitivity phenotype of *chl1-5* (Supplementary Fig. 10). Interestingly, the double homozygous line for *35S::NLP7-GFP* and the *nlp8-2* mutation, *NLP7-GFP nlp8-2*, failed to complement the *nlp8-2* mutation (Fig. 6a). This suggests that NLP7 and NLP8 have distinct functions from one another. To test whether the subcellular localization of NLP8 is under the control of nitrate like it is the case for NLP7, germinated seedlings of *35S::NLP8-GFP* and *35S::NLP7-GFP* were observed using confocal microscopy to examine subcellular localization of GFP signals with or without nitrate. The GFP signal of the NLP7-GFP was localized to the cytoplasm in the absence of nitrate, and with the application of nitrate became nuclear localized within a few minutes (Fig. 6b and Supplementary Movie 1), as reported in (ref. 22). In contrast, NLP8-GFP was localized in the nuclei regardless of nitrate application (Fig. 6b and Supplementary Movie 2). This suggests that nitrate may activate NLP8 by a different mechanism from that of NLP7.

The N-terminal region of NLP6 was shown to function as a nitrate-dependent transcriptional activation domain^20^. The N-terminal region (1–576 amino acids) of NLP8 was fused to the LexA DNA-binding domain (LexA_DB) to make the NLP8N-LexA_DB-YFP effector. The NLP8N-LexA_DB-YFP chimeric transcription factor construct was transformed together with the reporter, LexAOP-35Smini::GUS, into Col-0 plants (Fig. 6c). Similar to NLP8-GFP, NLP8N-LexA_DB-YFP also localized to the nuclei with or without nitrate application (Fig. 6b). Transgenic plants containing both chimeric constructs were examined for reporter expression. Seven-day-old nitrate-starved seedlings and imbibed seeds displayed nitrate-dependent GUS reporter expression (Fig. 6d and Supplementary Fig. 11). It is worth noting that, before application of nitrate, NLP8-YFP is localized in nuclei in the guard cells of cotyledons (Fig. 6b), and this cell type is competent (or contains components necessary) to trigger NLP8-dependent nitrate inducible gene expression (Fig. 6d). Taken together, these data indicate that the N-terminal region is responsible for induction of NLP8 by nitrate and NLP8-dependent nitrate signalling is unlikely to involve nitrate-dependent change in protein localization.

## Discussion

Nitrate is an important germination stimulator for many plant species, although its mode of action in seeds is poorly understood. This study identified NLP8 as a key regulator for nitrate signalling during germination. Transcriptome analysis indicates that NLP8 primarily activates the expression of nitrate-inducible transcription factors, and nitrogen metabolism enzymes (Fig. 3a), acting as a primary nitrate regulator to trigger secondary responses. Notably, time course expression analysis showed that NLP8-dependent nitrate induction is transient and is not seen in 12-h imbibed seeds (Fig. 3b,c), suggesting that a repressive mechanism comes into play to downregulate expression of NLP8-reglated genes in the latter stages of seed imbibition.

On the basis of our results, we propose that NLP8-dependent induction of *CYP707A2* regulates seed germination. The *cyp707a2* mutant displays reduced nitrate responsiveness, while nitrate-independent induction of *CYP707A2* in the *nlp8-2* mutant led to nitrate-independent germination (Fig. 4b), consistent with the notion that *CYP707A2* is an important downstream target of NLP8 in terms of germination control. The decrease of ABA by CYP707A2 is initiated in Phase I (ref. 26), which is well correlated with the temporal pattern of NLP8-dependent nitrate induction. The NLP8-dependent decrease of ABA is comparable to changes in ABA levels observed in light-regulated and temperature-regulated *Arabidopsis* seeds, suggesting that this difference in ABA levels is physiologically relevant^30^,^31^. We did not detect regulation of *NLP8* expression by nitrate (Supplementary Fig. 1), but activation of NLP8 signalling by nitrate induced the expression of *CYP707A2* (Figs 3 and 6). Cycloheximide-resistant gene expression is a characteristic feature that has been observed in Phase I (ref. 32), which is also consistent with the mode of NLP8 action. GA biosynthesis is activated in Phase II, which is associated with the initiation of germination^31^. It is known that the decrease in ABA levels is a prerequisite for the proper function of GA during *Arabidopsis* germination^2^. Therefore, it is likely that the NLP8-mediated induction of *CYP707A2* affects the germination control in Phase II, which includes the control of GA biosynthesis and sensitivity. In addition, it cannot be rule out that NLP8 influences seed germination via CYP707A2-independent pathway. Because some genes encoding transcription factors are nitrate-induced in an NLP8-dependent manner (Fig. 3a and Supplementary Fig. 4), it is possible that these transcription factors contribute to nitrate-promoted germination independently of CYP707A2. It is a future challenge to elucidate the secondary nitrate response triggered by NLP8 to fully understand nitrate-promoted seed germination.

The *nlp8* mutants display prominent phenotypes in nitrate-dependent germination. Nitrate-promoted seed germination of the *nia1nia2* mutant is NLP8-dependent (Fig. 2a), which indicates NLP8 acts in the nitrate signalling pathway to promote seed germination. The *chl1* mutant also displays nitrate insensitive germination^10^, but its phenotype is much milder than that of the *nlp8* mutant (Fig. 2b). In addition, only a negligible effect of the phosphor-mimic and phosphor-dead CHL1 mutant was observed on nitrate-promoted germination (Fig. 2b). This is consistent with the notion that CHL1 plays a minor role in the control of seed germination, and other nitrate sensor(s) might contribute to the NLP8-dependent germination control in the seeds.

We found 55 downregulated and 115 upregulated genes in the *nlp8-2* mutant independent of nitrate application (Supplementary Data 1). Downregulated genes in *nlp8-2* in the absence of nitrate induction include the highly nitrate-induced NLP8-dependent genes, such as *NIA2*, *CYP707A2* and *NIR1*. Because the *CYP707A2* transcript is downregulated in the dry seeds of *nlp8-2* (Fig. 3b), it is likely that NLP8 functions not only during germination, but also during seed development. On the other hand, upregulated genes in the *nlp8-2* mutant are enriched for those related to stresses, such as *thiredoxin-dependent peroxidase2* (*TPX2*) and *glutathione S-transferase1* (*GST1*) (Supplementary Fig. 6). It is possible that a defect in nitrate signalling induces a defense mechanism. In addition, nitrate-independent misexpressed genes in the *nlp8* mutants include *phytochrome interacting factor 3-like 5* (*PIL5*) and *abscisic acid-insensitive 4* (*ABI4*) (Supplementary Data 1 and Supplementary Fig. 6). Downregulation of these genes is expected to promote seed germination, which is opposite to the phenotype of *nlp8*, suggesting the possibility of feedback regulation in response to the defect in seed nitrate signalling.

Multiple environmental signals must coordinately interact with one another in the seed to modulate downstream events^1^. Transcriptome analysis in this study revealed potential crosstalk between nitrate and light signalling during germination. Two transcription factors related to light response, HY5-homolog (HYH) and far-red impaired responsive1 (FAR1)-family protein, are under the control of NLP8 (Fig. 3a and Supplementary Fig. 4). NLP8 was listed in the genes directly regulated by PIL5, a key regulator for light signalling during germination in *Arabidopsis*^33^. Interestingly, PIL5 is missexpressed in the *nlp8* mutant (Supplementary Fig. 6). PIL5 directly regulates GA signalling and indirectly regulates ABA and GA metabolism^34^. It is possible that crosstalk may occur between environmental signals and germination stimulants, to coordinately regulate seed dormancy and germination.

Nitrate promotion of seed germination was thought to be mediated by NO signalling^13^. However, our data presented in this study indicates that nitrate promotes germination by a distinct mechanism from that of NO signalling. The source of NO production is still unclear in plants, but it was reported that NO is produced by NO-associated protein 1 (NOA1) and NR in *Arabidopsis*. The *nia1nia2* mutant was still capable of responding to nitrate and promoted germination in an NLP8-dependent manner, despite the fact that this mutant has only 0.5% of the wild-type NR activity and lower endogenous NO levels^28^,^29^. This suggests that nitrate promotes germination independently of its assimilation products (Fig. 2a). In addition, NO-insensitive mutants, *prt6* and *ate1ate2,* are able to respond to nitrate during germination (Fig. 2a). This further supports that nitrate acts independently of NO signalling during seed germination.

Expression of NLP8 driven by the 35S promoter had no germination-promoting effect in the absence of nitrate (Fig. 6a) despite the fact that expression was two- to three-fold higher than wild type. This suggests that this level of overexpression itself has no obvious effect on the downstream events. However, it remains possible that this could be due to insufficient overexpression in particular cell or tissue types that would be required for a constitutive response. The *35S::NLP8-GFP* and *35S::NLP8N-LexA_DB-YFP* lines express GFP without nitrate application, suggesting that the NLP protein levels or its stability is not the primary regulation for NLP8 activation by nitrate. Post-translational activation of NLP6 and NLP7 functions were also reported. Overexpression of the NLP6-LexA construct did not transactivate the 8OP promoter, unless treated with nitrate^20^. Constitutive expression of NLP7 showed no additional effect except complementation of the poor growth phenotype of the *nlp7* mutant^19^, suggesting that NLPs require additional factors or post-translational modifications to regulate the primary response to nitrate.

**Figure 7:**
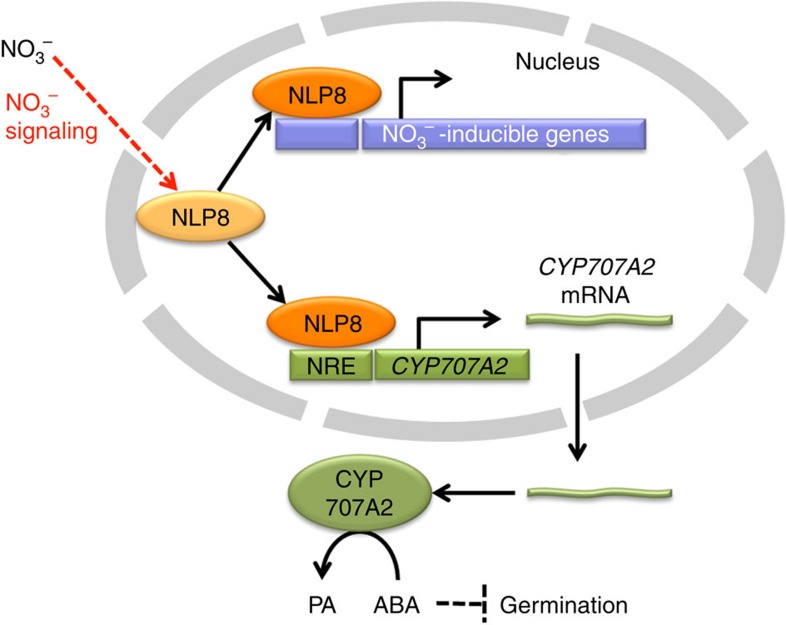
A proposed schematic model for NLP8 activity in regulating nitrate-promoted seed germination. NLP8 is located in nuclei regardless of nitrate application. Nitrate activates NLP8 post-translationally, which facilitates DNA binding to the NREs in the promoters of nitrate-regulated genes in seeds, including *CYP707A2*. Nitrate-induction of CYP707A2 decreases the ABA content, which promotes seed germination.

The localization or retention of NLP7 in nuclei is regulated by nitrate^22^. However, nitrate regulates the function of NLP8 differentially, since NLP8-GFP is localized to the nucleus in the absence of nitrate (Fig. 6b). It is noteworthy that stomata of cotyledons in which NLP8 is localized to the nucleus without nitrate treatment is competent to trigger an NLP8-dependent nitrate signalling (Fig. 6b,d). Therefore it is unlikely that nitrate-independent nuclear localization of NLP8 is due to the components of missing nitrate signalling caused by ectopic expression. In addition, another new finding is that binding of NLP8 to the *CYP707A2* promoter *in vivo* is nitrate-dependent, even though NLP8 is localized to the nucleus before nitrate application (Figs 5c and 7). The inability of NLP7-GFP to complement *nlp8-2* also supports the notion that NLP8 and NLP7 demonstrate distinct differences in regulation, due to differing functions. It has been argued that mechanisms of plant nitrate responses vary depending on developmental and environmental contexts, which is called ‘matrix effect^35^. Our findings indicate that multiple mechanisms that activate NLP functions form an essential component of complex nitrate responses in plants.

## Methods

### Plant material and growth conditions

*nlp8-1* (SALK_031064), *nlp8-2* (SALK_140298), *nlp8-3* (WiscDsLoxHs201_10C), *nlp8-4* (SALK_026238), *nlp9-1* (SALK_025839) and *nlp9-2* (SALK_098057) were obtained from ABRC. *nlp8-5* (FLAG_537A05) and Ws-4 were ordered from INRA. *chl1-5*, *T101A/chl1-5* and *T101D/chl1-5* (ref. 16), *ate1ate2* and *prt6* (ref. 15), *35S::NLP7-GFP*^19^, *nia1nia2* (*nia1-1nia2-5*)^29^, *aba2* (*aba2-2*)^36^, *cyp707a1* (*cyp707a1-1*)^4^ and *cyp707a2* (*cyp707a2-1*)^5^ were described previously. All lines in Col-0 background were grown at 22 °C until flowering and then switched to 16 °C and continuous light for seed maturation. *nlp8-5*, Ws-4, *nlp8-2*/Cvi and Cvi were grown at 22 °C. For ChIP and microscope analysis, seeds were surface sterilized and stratified for 4 days and then sown on nitrate-free 1/2 MS medium containing 3 mM KCl (referred here as KCl plate) or KNO_3_ (referred here as KNO_3_ plate)^22^, for indicated periods of time.

### Germination test

Seeds stored for 2 weeks to 3 months at room temperature were used for germination test. Approximately 50 seeds were soaked in 1 ml liquid media in a 24-well plate. Plates were incubated for 7 days at 23 °C under continuous white light condition (25 μmol m^−2^ s^−1^). Radicle protrusion was used as a criterion for germination.

### Constructs and transformation

For *35S::NLP8-GFP*, full-length CDS of NLP8 was amplified from cDNA synthesized from Col-0 seeds and cloned into the binary vector pGWB505 (ref. 37). Transcript levels of *NLP8* in these transgenic lines were examined by qRT–PCR (Supplementary Fig. 12). To generate *35S::CYP707A2* lines, *CYP707A2* cDNA containing 5′- and 3′-UTR were PCR amplified, cloned into pENTR/D/TOPO vector (Thermo Fisher Scientific) and subsequently cloned into the modified binary vector pGWB2, which contained the enhancer sequence and 35S promoter of pBE2113N between HindIII and XbaI sites^37^,^38^. For A2-GUS and promoter deletions, different lengths of the promoter region of *CYP707A2* were amplified from Col-0 genomic DNA and cloned into the binary vector pMDC162 (ref. 39). For the transient assay, deletion and mutated *CYP707A* promoters were cloned into the binary vector pGWB535 (ref. 37). Mutated DNA fragment was generated by PCR-mediated site-directed mutagenesis. For the chimeric construct, NLP8N and LexA_DB was first amplified from *35S::NLP8-GFP* and pER8 (ref. 40), respectively. NLP8N-LexA_DB translational fusion was amplified by overlap PCR, and cloned into binary vector pEarlygate101 (ref. 41). LexAOP-35Smini was amplified directly from pER8 and cloned into pMDC162. All constructs were sequence verified and transformed into Col-0 using the floral dip method. Primers are listed in Supplementary Data 2.

### Protoplast transient assay

A luciferase (LUC) reporter activity driven by deletion and mutated *CYP707A2* promoters were tested in a protoplast transient assay. *35S::NLP8-GFP* and *35S::GUS* (pCAMBIA1105.1R) were used as an effector and an internal control plasmid, respectively. Transfection-grade plasmids were isolated by FastGene Xpress Plasmid PLUS Kit (FastGene). *Arabidopsis* protoplasts were prepared from young rosette leaves of 3-week-old plants as previously described^42^. A reporter (7.5 μg), an effector (7.5 μg) and an internal control GUS plasmid (1 μg) were co-transfected into ∼2 × 10^4^ protoplasts by a 20% w/v polyethylene glycol 4000-mediated method. In the non-effector experiment, the pMD20 (7.5 μg) plasmid containing ACT2 (AT3G18780) cDNA was used to adjust total amount of input DNA as a control. After transfection, cells were cultured with 0.5 M mannitol and 4 mM MES (pH 5.7) containing 20 mM KCl or KNO_3_ for 18 h at 22 °C. Cells were collected, and then LUC and GUS activities were measured using luciferase assay system kit (Promega) and 4-methylumbelliferyl-D-glucuronide (Sigma), respectively, as substrates. The relative promoter activity was determined by calculating ratios of LUC and GUS activities.

### Expression analysis and ChIP

RNA was extracted from seeds imbibed in 1 mM KCl or KNO_3_ for 6 h. Briefly, total RNA was extracted by acid phenol extraction, precipitated with lithium chloride and DNase digested as previously described^43^. After DNA digestion, cDNA was synthesized using RevertAid First Strand cDNA Synthesis Kit (Life Technologies). Paired-end RNA-seq was performed on Illumina GAIIx. qRT-PCR was performed using SsoFast EvaGreen supermixes (Bio-Rad) and a CFX96 Real-Time PCR Detection System (Bio-Rad). At1g13320 was used as the reference gene^44^,^45^. The biological replicates analysed were as follows: A2-GUS #1, *n*=3; A2-GUS #6, *n*=3; d1-GUS #3, *n*=4; d2-GUS #2, *n*=3; d2-GUS #6, *n*=5; d3-GUS #1, *n*=3; d3-GUS #11, *n*=3. The expression stability of this gene was confirmed by geNORM analysis (Supplementary Fig. 13)^46^. ChIP experiment was performed as the method described in Saleh *et al*.^47^ with minor modifications. Ten-day-old *35S::NLP8-GFP* was grown on 3 mM KNO_3_ plates and then transferred to nitrate-free 1/2 MS liquid medium containing 3 mM KCl for 3 days, then resupplied with 10 mM KNO_3_ for 20 min. Approximately 0.5 g of seedlings were treated with the cross-linking buffer containing 1% formaldehyde, 0.4 M sucrose, 10 mM Tris-HCl (pH 8), 1 mM PMSF, 1 mM EDTA and 10 mM KNO_3_ for 10 min. After sonication, DNA was recovered from the sample incubated with anti-GFP (A11122, Life Technologies) and Protein G Agarose Beads (#9007, Cell Signaling Technology). Experiments were done in four replicates and relative fold enrichment was calculated by normalizing the amount of a target DNA fragment against that of no antibody control.

### Electrophoretic mobility shift assay

The RWP-RK DNA binding domain (amino acid 549–686) of NLP8 was PCR amplified and cloned into pET32a-LIC. Protein expression was induced by application of 1 mM IPTG to BL21 carrying pET32a-NLP8 when OD600 was 0.4, and incubated at 16 °C overnight. Protein was purified using HisPur Ni-NTA Spin Column (Thermo Fisher Scientific) according to the manual. For preparing probes and competitors, single-strand oligonucleotides were annealed to form dsDNA. Two copies of tandem fragments were obtained through self-ligation after T4 polynucleotide kinase treatment. Probes and competitors were amplified by PCR using biotin labeled or non-labeled primers. EMSA was performed using LightShift Chemiluminescent EMSA Kit (Thermo Fisher Scientific) and membrane was exposed to ChemiDoc^TM^ MP Imaging System (Bio-Rad). Around 1.5 ng biotin labelled probe and 200 ng NLP8 protein were used for each reaction. Competitors were 50-fold (+) or 100-fold (++).

### CAPS marker design

The genome-wide SNP data of Col-0 and Cvi-0 was obtained from the AtSNPtile1 SNP datasets previously published^48^,^49^. The array information was updated to version TAIR10 based on the AtSNPtile1 probe sequences (http://aquilegia.uchicago.edu/naturalvariation/cisTrans/ArrayAnnotation.html) and TAIR10 sequence datasets (ftp://ftp.arabidopsis.org/home/tair). Pseudo BLAST alignments were constructed using this data set and TAIR10 genome sequence by replacing the bases in TAIR10 sequence by SNPs and formatting the alignment to match the BLAST output format. BlastDigester was run on these alignments after setting the ‘show only differential cutters' option^50^. The output for BlastDigester was kept if DNA could be digested with the following enzymes: AccI, BamHI, BclI, BglI, BglII, SnaBI, EcoRV, EcoRI, HindIII, KpnI, NaeI, NdeI, NcoI, PstI, PvuII, SalI, SacI, SmaI, SpeI, XbaI and XhoI. CAPS markers are listed in Supplementary Data 2.

### RNA-seq data processing

RNA-seq reads were aligned to *Arabidopsis thaliana* gene models (TAIR 10) using the short read mapping tool, novoalign (novocraft.com). The reads mapped to multiple locations were discarded. The reads mapped to each gene were counted and rpkm (reads per kilobase per million) was calculated using an in house php script. Differential gene expression was analysed with the R package DEGseq^51^. The replicates were compared by method FC (fold-change) with a cutoff of abs(log2(FC))<1.3219 for all replicate groups. The treatments (KCl versus KNO_3_) were compared by method MARS (MA-plot-based method with Random Sampling model) with a cutoff of *P*<0.001 for both Col-0 and *npl8-2*. A gene is differentially expressed between treatments only when its *P* value is below 0.001 and no significant change between the replicates is observed. RNA-seq set is presented in Supplementary Data 1. For making heatmap, genes differentially expressed by nitrate treatment at greater than two-fold change in Col-0 were selected to generate heatmap using GENE-E (http://www.broadinstitute.org/cancer/software/GENE-E/index.html). Overrepresentation of particular GO terms in nitrate-induced genes was judged by *P* values of the hypergeometric distribution, calculated as *P*=BC(*M*, *x*) × BC(*N*−*M*, *n*−*x*) BC(*N*, *n*)^−1^ with BC as the binominal coefficient, *x* as the number of nitrate-induced genes belonging to a particular GO category, *n* as the total number of nitrate-induced genes, *M* as the number of genes belonging to a particular GO category, and with *N* as the total number of genes in the *Arabidopsis* genome^52^.

### Quantification of ABA contents

Dry seeds and imbibed seeds were collected and homogenized in 80% (v/v) methanol containing 1% (v/v) glacial acetic acid by TissueLyser (Qiagen). The internal standard was added and stored overnight at 4 °C.

Samples were centrifuged to remove debris, and the pellet was washed twice. The supernatant was evaporated in a SpeedVac, reconstituted in 1 ml of 1% (v/v) acetic acid. ABA and d_6_-ABA were purified by solid phase extraction using Oasis HLB, MCX and WAX cartridge columns (Waters) as previously described^26^. The solvent was removed under vacuum and subjected to the LC-ESI-MS/MS analysis (Agilent 6,410 TripleQuad LC/MS system). An LC (Agilent 1200 series) equipped with a 50 × 2.1 mm, 1.8-μm Zorbax SB-Phenyl column (Agilent) was used with a binary solvent system comprising 0.01% (v/v) acetic acid in water (Solvent A) and 0.05% (v/v) acetic acid in acetonitrile (Solvent B). Separations were performed using a gradient of increasing acetonitrile content with a flow rate of 0.2 ml min^−1^. The gradient was increased linearly from 3% B to 50% B over 15 min. The retention time of ABA was 14.0 min. MS/MS conditions were as follows: capillary 4.0 kV; source temperature, 100 °C; desolvation temperature, 350 °C; cone gas flow, 0 l min^−1^; desolvation gas flow, 12 l min^−1^; fragmentor, 140; collision energy, 8; cell accelerator voltage, 7; polarity, negative; MS/MS transition, 269/159 *m*/*z* for d_6_-ABA and 263/153 *m*/*z* for ABA. A calibration curve was made with d_6_-ABA and ABA.

### Microscope imaging

Five-day-old *35S::NLP7-GFP* or *35S::NLP8-GFP* lines were grown on KNO_3_ plates and then transferred to KCl plate for 2 days. Nitrate was resupplied by transferring seedlings to filter paper soaked in nitrate-free 1/2 MS liquid medium containing 3 mM KCl or KNO_3_ for 20 min. Cotyledons were mounted in 40 μl 3 mM KCl or KNO_3_ and visualized with a Leica TCS SP5 Confocal microscope.

For the GUS staining, seven-day-old seedlings grown on KCl plates were transferred to filter paper and soaked in nitrate-free 1/2 MS liquid medium containing 10 mM KCl or KNO_3_ for 4 h or seeds were imbibed in 10 mM KCl or KNO_3_ for 18 h before GUS staining. The seedlings were imaged using the Olympus SZX7 stereo microscope, and the embryos were imaged using the Olympus BX51 Differential Interference Contrast microscope. For movies, plants were cultivated on 2.5 mM ammonium succinate for 7 days in long day, 3 mM nitrate was then added under the Zeiss LSM 710 Confocal. Pictures were taken every 3 s and combined to export as movie using ZEN 2012 lite software.

### Data availability

RNA-seq data generated as part of this study have been deposited in the NCBI SRA database under accession code SRP082409. The authors declare that all other data supporting the findings of this study are available within the paper and its Supplementary Files or are available from the corresponding author upon request.

## Additional information

**How to cite this article:** Yan, D. *et al*. NIN-like protein 8 is a master regulator of nitrate-promoted seed germination in *Arabidopsis*. *Nat. Commun.* **7,** 13179 doi: 10.1038/ncomms13179 (2016).

## Supplementary Material

Supplementary Information Supplementary Figures 1 - 13

Supplementary Data 1 RNA-seq expression data for Col-0 and nlp8-2 seeds imbibed for 6-h in the presence of 1 mM KCl or KNO3.

Supplementary Data 2 Primers used in this study.

Supplementary Movie 1 Localization of NLP7-GFP in response to nitrate. Confocal imaging was performed on NLP7-GFP plants grown on 2.5 mM ammonium succinate for 7 days. Pictures was recorded every 3 seconds after addition of 3 mM nitrate.

Supplementary Movie 2 Localization of NLP8-GFP in response to nitrate. Confocal imaging was performed on NLP8-GFP plants grown on 2.5 mM ammonium succinate for 7 days. Pictures was recorded every 3 seconds after addition of 3 mM nitrate.
