# Supplementary material for: NIN-like protein 8 is a master regulator of nitrate-promoted seed germination in Arabidopsis
Source: Nat Commun. 2016 Oct 12;7:13179. doi: 10.1038/ncomms13179 (PMC5064020; doi:10.1038/ncomms13179)
Supplement: Supplementary Information — Supplementary Figures 1 - 13 [file ncomms13179-s1.pdf]

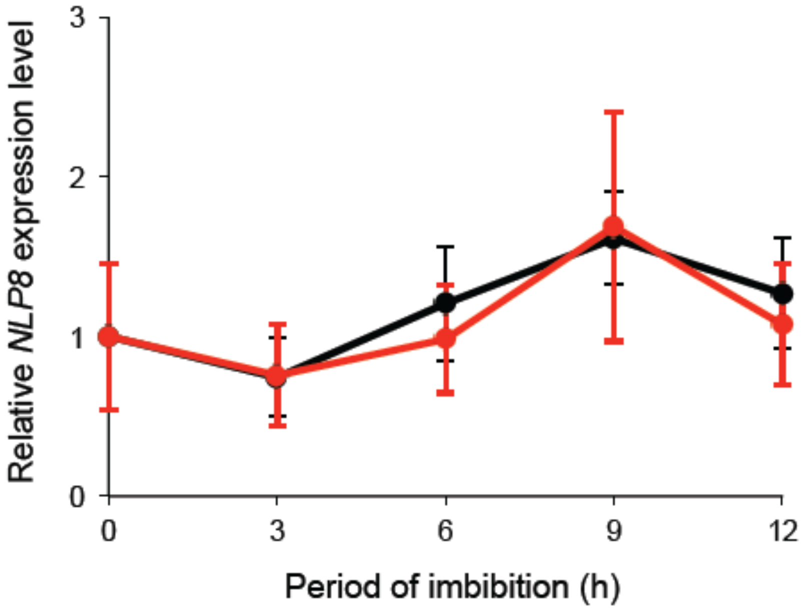

Supplementary Figure 1 | qRT-PCR expression analysis of *NLP8* with and without KNO_3 during germination.

Seeds of Col-0 were harvested from plants grown at 16°C, stored for 2 months, imbibed for indicated time periods in water with 1 mM KCl (black line) or KNO_3 (red line). qRT-PCR was performed with three independent biological replicates. Expression levels were normalized by that of At1g13320. Averages of relative expression are shown with SD (n=3).

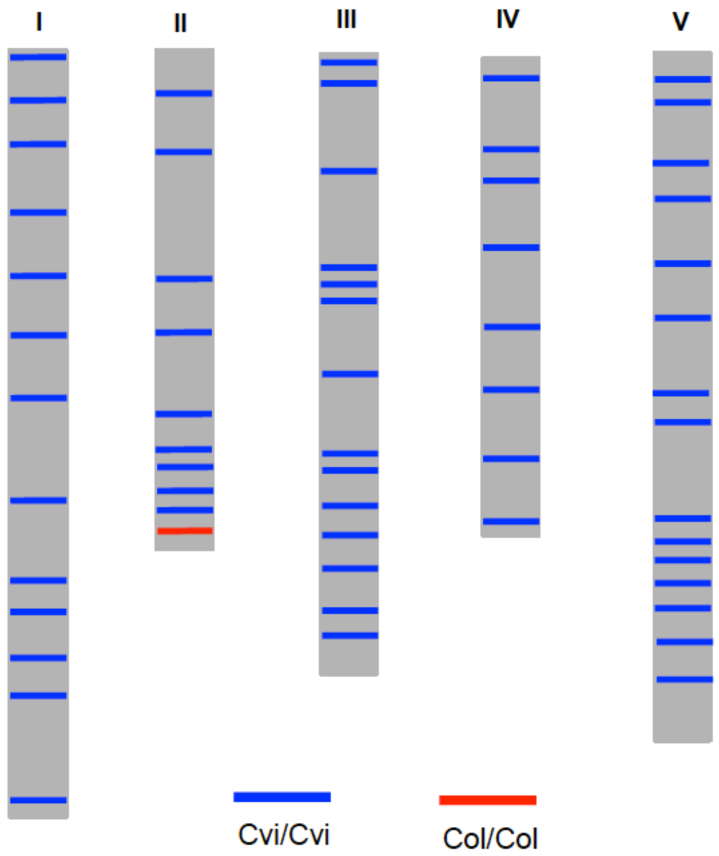

Supplementary Figure 2 | Construction of the near isogenic line, *nlp8-2*/Cvi.

Location of genetic markers is shown on the five Arabidopsis chromosomes (From I to V). The *nlp8-2* mutant in Col background was crossed to Cvi four times to obtain the *nlp8-2* mutant in Cvi background. The *nlp8-2*/Cvi line was confirmed that all tested markers, except for NLP8, showed the Cvi/Cvi homozygote. The *nlp8-2* locus is indicated by red, while other genetic markers are indicated by blue. The method to create genetic markers distinguishing between Col and Cvi genomic regions is described in Methods, and marker information is in Supplementary Data 2.

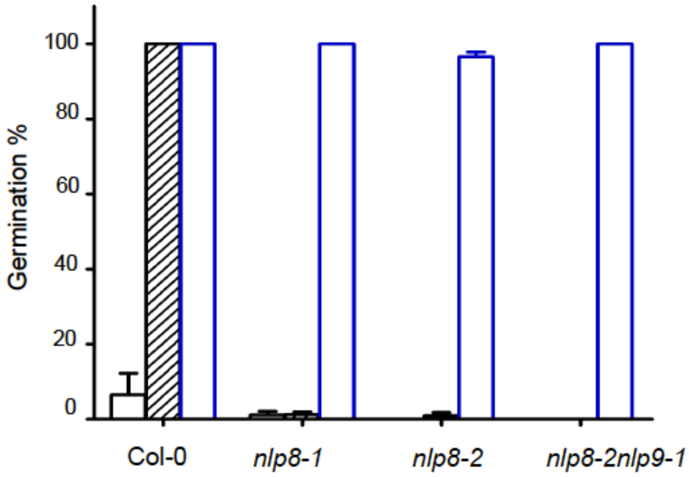

Supplementary Figure 3 | The effect of stratification on germination of the *nlp8* mutant.

Seeds of Col-0, *nlp8-1*, *nlp8-2*, *nlp8-2nlp9-1* mutants were harvested from plants grown at 16°C, stored for 2 months. Germination test was performed using biological triplicates, and radicle protrusion through endosperm was counted 7 days after sowing. Percentage of germination in water with KCl without stratification (white bar), with KNO_3 without stratification (lined bar), and with KCl with stratification for 4 days (blue bar) is shown with SD (*n*=3).

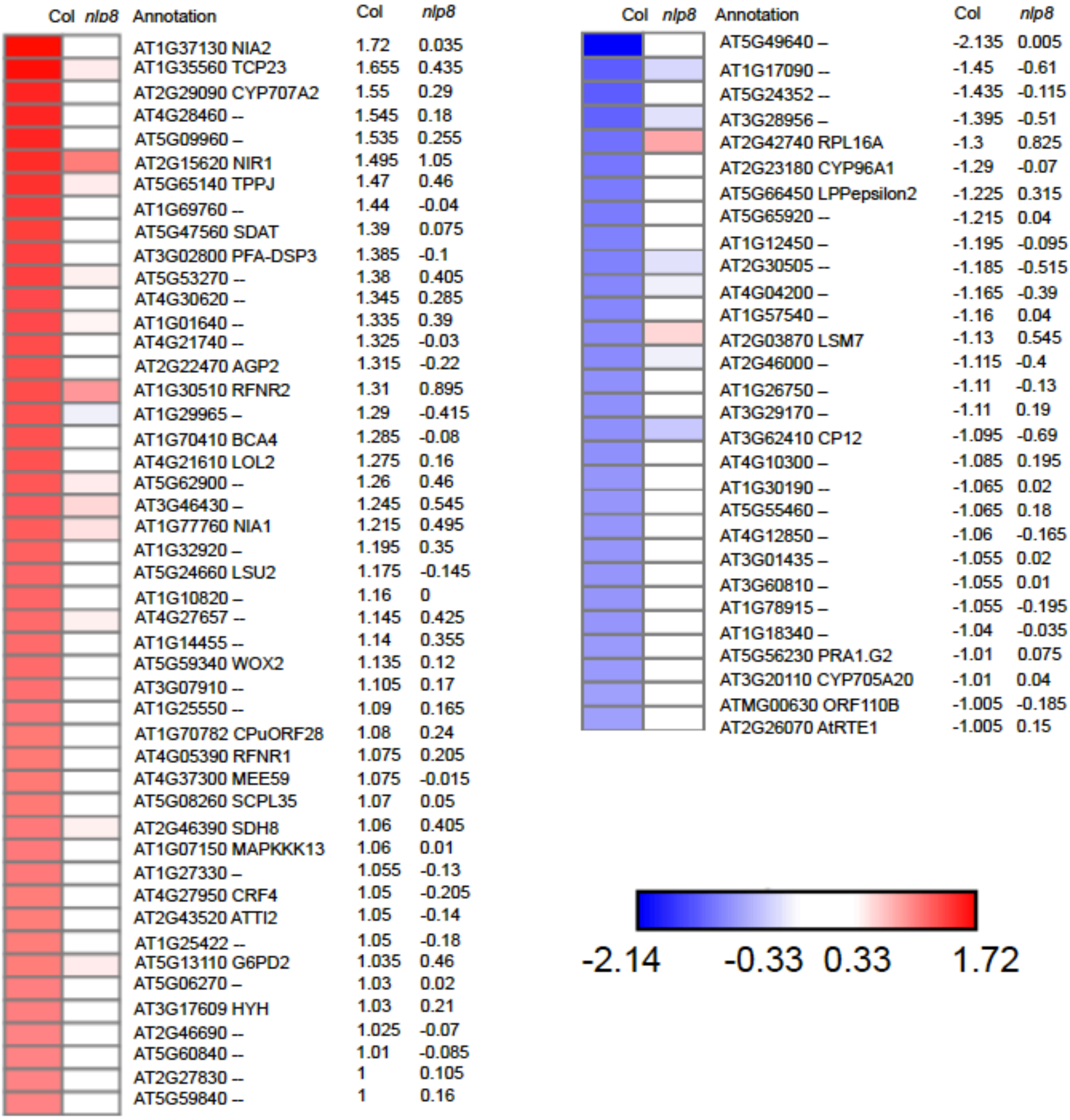

Annotation	Col	*nlp8*
AT1G37130 NIA2	1.72	0.035
AT1G35560 TCP23	1.655	0.435
AT2G29090 CYP707A2	1.55	0.29
AT4G28460 –	1.545	0.18
AT5G09960 –	1.535	0.255
AT2G15620 NIR1	1.495	1.05
AT5G65140 TPPJ	1.47	0.46
AT1G69760 –	1.44	-0.04
AT5G47560 SDAT	1.39	0.075
AT3G02800 PFA-DSP3	1.385	-0.1
AT5G53270 –	1.38	0.405
AT4G30620 –	1.345	0.285
AT1G01640 –	1.335	0.39
AT4G21740 –	1.325	-0.03
AT2G22470 AGP2	1.315	-0.22
AT1G30510 RFNR2	1.31	0.895
AT1G29965 –	1.29	-0.415
AT1G70410 BCA4	1.285	-0.08
AT4G21610 LOL2	1.275	0.16
AT5G62900 –	1.26	0.46
AT3G46430 –	1.245	0.545
AT1G77760 NIA1	1.215	0.495
AT1G32920 –	1.195	0.35
AT5G24660 LSU2	1.175	-0.145
AT1G10820 –	1.16	0
AT4G27657 –	1.145	0.425
AT1G14455 –	1.14	0.355
AT5G59340 WOX2	1.135	0.12
AT3G07910 –	1.105	0.17
AT1G25550 –	1.09	0.165
AT1G70782 CPuORF28	1.08	0.24
AT4G05390 RFNR1	1.075	0.205
AT4G37300 MEE59	1.075	-0.015
AT5G08260 SCPL35	1.07	0.05
AT2G46390 SDH8	1.06	0.405
AT1G07150 MAPKKK13	1.06	0.01
AT1G27330 –	1.055	-0.13
AT4G27950 CRF4	1.05	-0.205
AT2G43520 ATTI2	1.05	-0.14
AT1G25422 –	1.05	-0.18
AT5G13110 G6PD2	1.035	0.46
AT5G06270 –	1.03	0.02
AT3G17609 HYH	1.03	0.21
AT2G46690 –	1.025	-0.07
AT5G60840 –	1.01	-0.085
AT2G27830 –	1	0.105
AT5G59840 –	1	0.16

Annotation	Col	*nlp8*
AT5G49640 –	-2.135	0.005
AT1G17090 –	-1.45	-0.61
AT5G24352 –	-1.435	-0.115
AT3G28956 –	-1.395	-0.51
AT2G42740 RPL16A	-1.3	0.825
AT2G23180 CYP96A1	-1.29	-0.07
AT5G66450 LPPepsilon2	-1.225	0.315
AT5G65920 –	-1.215	0.04
AT1G12450 –	-1.195	-0.095
AT2G30505 –	-1.185	-0.515
AT4G04200 –	-1.165	-0.39
AT1G57540 –	-1.16	0.04
AT2G03870 LSM7	-1.13	0.545
AT2G46000 –	-1.115	-0.4
AT1G26750 –	-1.11	-0.13
AT3G29170 –	-1.11	0.19
AT3G62410 CP12	-1.095	-0.69
AT4G10300 –	-1.085	0.195
AT1G30190 –	-1.065	0.02
AT5G55460 –	-1.065	0.18
AT4G12850 –	-1.06	-0.165
AT3G01435 –	-1.055	0.02
AT3G60810 –	-1.055	0.01
AT1G78915 –	-1.055	-0.195
AT1G18340 –	-1.04	-0.035
AT5G56230 PRA1.G2	-1.01	0.075
AT3G20110 CYP705A20	-1.01	0.04
ATMG00630 ORF110B	-1.005	-0.185
AT2G26070 AtRTE1	-1.005	0.15

Supplementary Figure 4 | Nitrate-regulated genes in 6-hour imbibed seeds in Col-0 or *nlp8-2* mutant.

Seeds were imbibed in water with 1mM KCl or KNO_3 for 6 hours and RNA were extracted for RNA-seq. The readouts from KNO_3-treated seeds were normalized by those from KCl control and a mean of two biological repeats was used to generate a heatmap.

ABA metabolism and signaling

Gene	Locus	Col				*nlp8*			
		KCl		KNO_3		KCl		KNO_3	
		Exp1	Exp2	Exp1	Exp2	Exp1	Exp2	Exp1	Exp2
ABA1/ZEP	At5g67030	194	153	174	189	204	186	208	205
ABA4	At1g67080	3	2	3	2	3	2	2	3
NCED2	At4g18350	2	2	2	2	6	5	5	6
NCED3	At3g14440	1	1	1	1	1	1	1	2
NCED5	At1g30100	14	16	18	18	9	7	9	7
NCED6	At3g24220	2	3	2	2	2	2	2	2
NCED9	At1g78390	6	6	7	8	8	8	8	9
ABA2/SDR	At1g52340	9	5	7	8	9	9	8	8
AAO3	At2g27150	6	4	5	5	7	6	7	7
ABA3	At1g16540	16	13	15	16	22	19	21	24
CYP707A1	At4g19230	4	3	3	3	6	6	5	5
CYP707A2	At2g29090	73	65	198	205	10	7	12	9
CYP707A3	At5g45340	6	6	6	8	8	10	7	9
CYP707A4	At3g19270	1	1	1	1	1	1	1	1
PYR1/RCAR11	At4g17870	26	19	28	30	29	39	25	39
PYL1/RCAR12	At5g46790	2	2	1	2	2	1	2	1
PYL2/RCAR14	At2g26040	16	32	47	40	15	27	19	27
PYL3/RCAR13	At1g73000	1	1	1	1	1	2	2	2
PYL4/RCAR10	At2g38310	6	10	6	10	1	2	2	1
PYL6/RCAR9	At2g40330	1	1	1	1	1	1	1	2
PYL7/RCAR2	At4g01026	39	9	17	15	47	29	44	45
PYL9/RCAR1	At1g01360	13	3	7	4	12	11	10	14
PYL8/RCAR3	At5g53160	7	1	3	2	3	4	4	4
PYL11/RCAR5	At5g45860	20	16	13	12	11	9	11	3
PYL12/RCAR6	At5g45870	13	2	9	6	15	11	12	13
PYL13/RCAR7	At4g18620	9	9	10	11	14	11	13	15
ABI1	At4g26080	11	7	8	9	12	15	10	12
ABI2	At5g57050	30	12	17	16	37	31	33	35
HAB1	At1g72770	37	27	35	35	65	70	65	70
HAB2	At1g17550	33	22	27	25	35	31	33	36
AHG1	At5g51760	671	955	1017	1017	626	695	671	809
AHG3/AtPP2CA	At3g11410	64	34	40	41	65	60	58	60
HAI1	At5g59220	6	3	4	3	13	13	10	13
HAI3	At2g29380	103	74	79	77	83	73	89	72
HAI2/AIP1/HON	At1g07430	103	123	97	95	67	67	57	60
SnRK2.2/SRK2D	At3g50500	73	42	63	56	75	70	77	75
SnRK2.3/SRK2I	At5g66880	72	56	58	61	43	39	45	41
SnRK2.6/SRK2E/(	At4g33950	17	28	35	35	29	34	29	40
ABI3	At3g24650	100	175	125	151	89	92	85	102
ABI4	At2g40220	14	23	29	29	7	5	6	6
ABI5	At2g36270	434	512	519	465	391	422	367	375

GA metabolism and signaling

Gene	Locus	Col				*nlp8*			
		KCl		KNO_3		KCl		KNO_3	
		Exp1	Exp2	Exp1	Exp2	Exp1	Exp2	Exp1	Exp2
AtCPS/GA1	At4g02780	1	1	1	1	1	1	1	1
AtKS/GA2	At1g79460	7	6	6	6	7	4	6	5
AtKO/GA3	At5g25900	160	91	108	103	140	106	136	122
AtKAO1	At1g05160	3	4	3	3	4	4	5	4
AtKAO2	At2g32440	13	10	11	12	7	6	8	7
AtGA20ox1	At4g25420	1	1	1	1	1	1	1	1
AtGA20ox2	At5g51810	1	1	1	1	1	1	1	1
AtGA20ox3	At5g07200	9	11	5	11	3	1	2	2
AtGA20ox4	At1g60980	1	1	1	1	1	1	1	1
AtGA20ox5	At1g44090	1	1	1	1	1	1	1	1
AtGA3ox1	At1g15550	1	1	1	1	1	1	1	1
AtGA3ox2	At1g80340	1	1	1	1	1	1	1	1
AtGA3ox3	At1g80330	1	1	1	1	1	1	1	1
AtGA2ox1	At1g78440	1	1	1	1	1	1	1	1
AtGA2ox2	At1g30040	4	3	3	3	8	5	5	6
AtGA2ox3	At2g34555	1	1	1	1	1	1	1	1
AtGA2ox4	At1g47990	1	1	1	1	1	1	1	1
AtGA2ox6	At1g02400	6	6	5	6	8	10	9	9
GAMT1	At4g26420	1	1	1	1	1	1	1	1
GAMT2	At5g56300	1	1	1	1	1	1	1	1
AtGID1A	At3g05120	114	86	93	97	78	60	89	84
AtGID1B	At3g63010	1	1	1	1	1	1	1	1
AtGID1C	At5g27320	32	10	20	18	23	19	24	19
RGA1	At2g01570	21	18	16	18	13	13	12	11
RGL1	At1g79380	19	25	20	27	20	21	20	22
RGL2	At3g03450	17	10	14	13	8	7	9	8
RGL3	At5g17490	8	6	6	7	5	3	5	4
SLY1	At4g24210	15	16	24	18	12	12	12	10
SPY	At3g11540	39	25	31	30	42	34	41	41

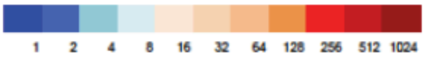

Supplementary Figure 5 | Expression of ABA- and GA-related genes in 6-hour imbibed Col-0 and *nlp8* mutant seeds.

The numbers in the heatmap indicate the expression values from the RNA-seq data.

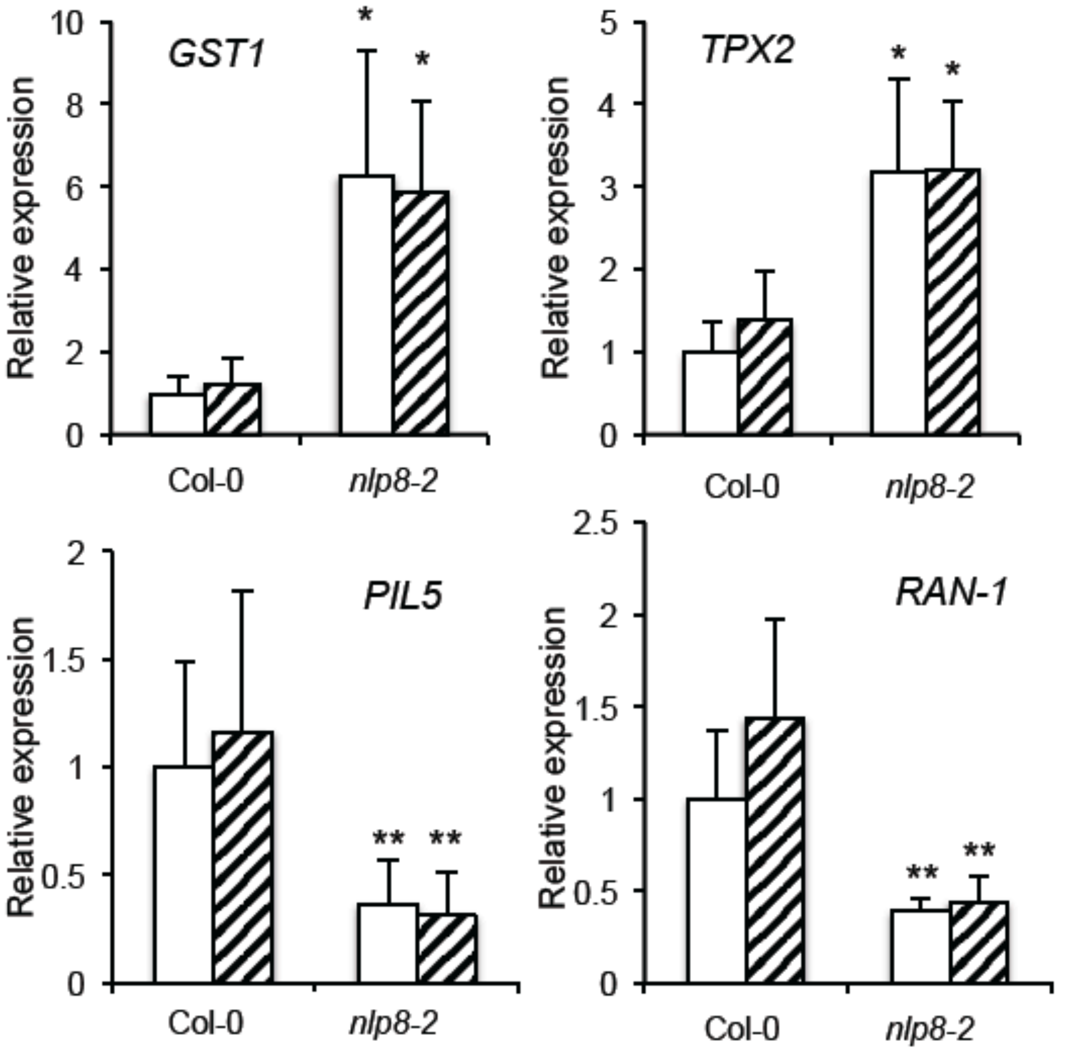

Supplementary Figure 6 | qRT-PCR expression analysis of misexpressed genes in 6-hour imbibed *nlp8* mutant seeds

Seeds of Col-0 and *nlp8-2* mutant were harvested from plants grown at 16°C, stored for 2 months, imbibed for 6 hours in water with 1 mM KCl (white bar) or KNO_3 (lined bar). qRT-PCR was performed with four biological replicates. Expression levels were normalized by that of At1g13320. Averages of relative expression relative to those in Col-0 seeds treated with KCl are shown with SD (n=4). * and ** indicate P <0.05 and P <0.01 (Student's *t*-test), respectively.

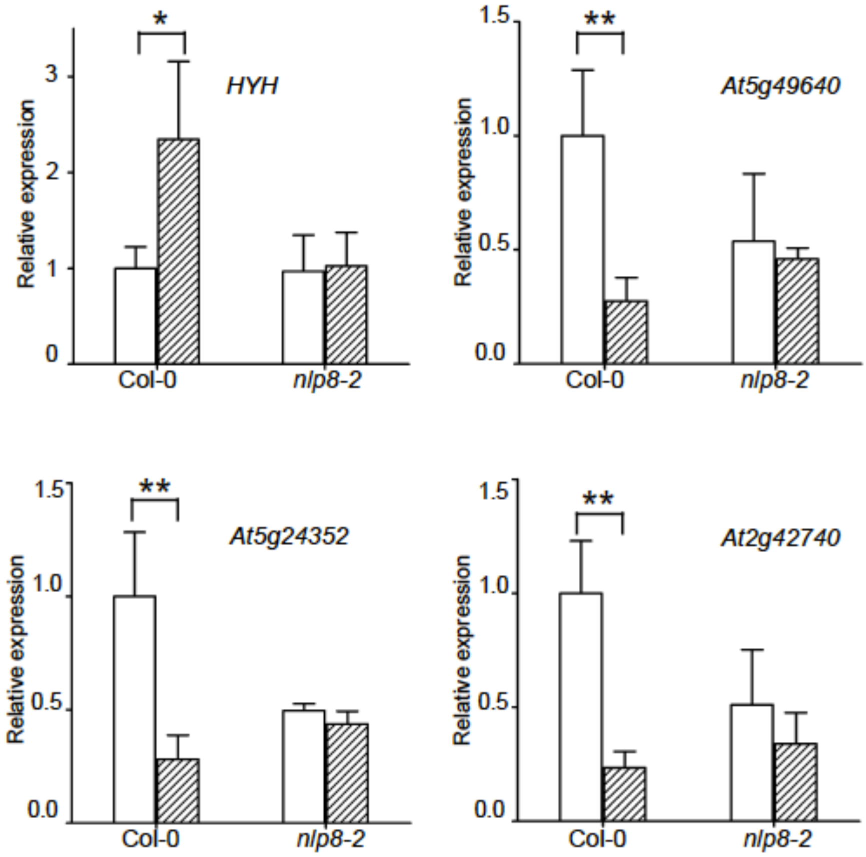

Supplementary Figure 7 | qRT-PCR expression analysis of nitrate responsive genes in 6-hour imbibed Col-0 and *nlp8* mutant seeds.

Seeds of Col-0 and *nlp8-2* mutant were harvested from plants grown at 16°C, stored for 2 months, imbibed for 6 hours in water with 1 mM KCl (white bar) or KNO_3 (lined bar). qRT-PCR was performed with three independent biological replicates. Expression levels were normalized by that of At1g13320. Averages of relative expression of nitrate up-regulated gene (HYH) and down-regulated genes (At5g49640, At5g24352, and At2g42740) are shown with SD (n=3). * and ** indicate $P < 0.05$ and $P < 0.01$ (Student's *t*-test), respectively.

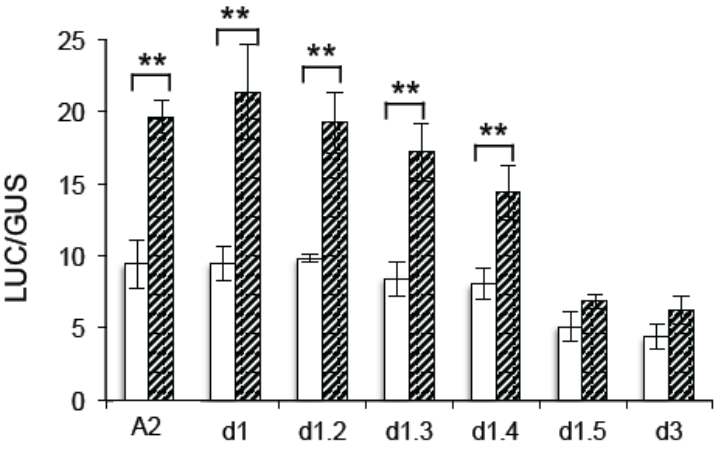

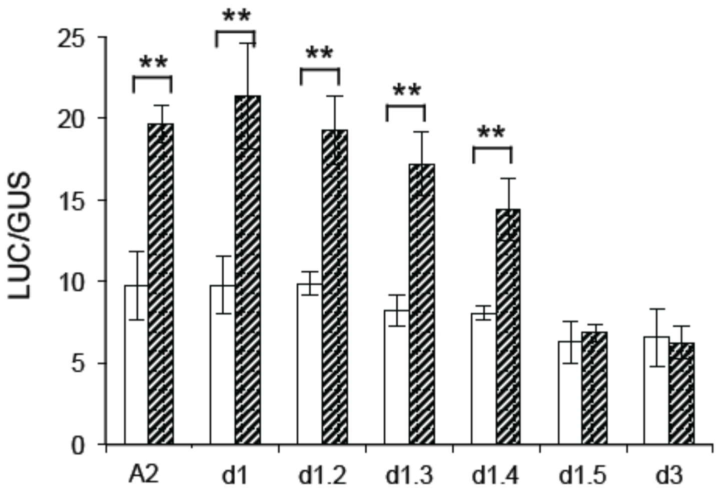

Supplementary Figure 8 | Promoter deletion analysis to identify the region responsible for NLP8-dependent gene expression

The deletion series of *CYP707A2* promoters were fused to the LUC reporter gene, and the promoter activities were examined in a protoplast system. The top graph indicates the promoter activities of deleted *CYP707A2* promoters when transiently expressing *NLP8* under KCl treatment (white bar) and nitrate treatment (lined bar), while the bottom graph indicates the promoter activities transiently expressing NLP8 (lined bar) and the control ACT2 (white bar) under nitrate treatment. The promoter length of each construct is as follows: A2, 1887-bp; d1, 1661-bp; d1.2, 1638-bp; d1.3, 1615-bp; d1.4, 1589-bp; d1.5, 1549-bp; d3, 741-bp. 35S:GUS construct was co-introduced with NLP8 or ACTIN2, and the LUC activities were normalized by GUS activities. Three independent experiments were performed, and averages of relative expression are shown with SD. ** indicates the P value <0.01 by Student's *t*-test.

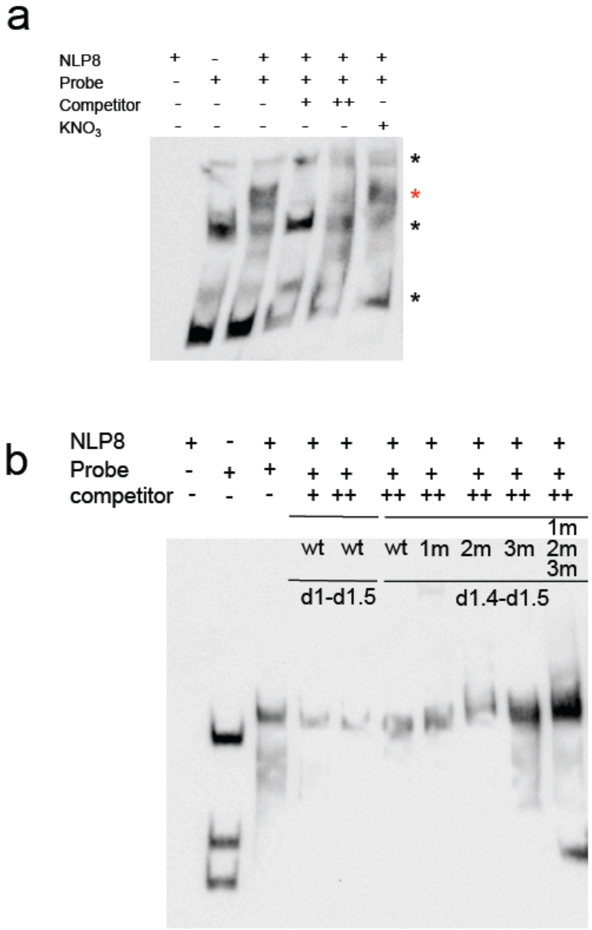

Supplementary Figure 9 | The RWP-RK domain of NLP8 binds to the NRE of the CYP707A2 promoter.

(a) A biotin-labeled probe containing 2 copies of d1-d1.5 (1.5ng) and RWP-RK domain of NLP8 (200 ng) were used for each EMSA reaction and separated in a 5% native PAGE gel. Competitors were 2 copies of d1-d1.5 fragment without biotin labeling. KNO3 was added to 10 mM as a final concentration. A red star indicates the shifted band, while the black stars indicate the signal from free probe. (b) The full image of the blot shown in Fig. 5f.

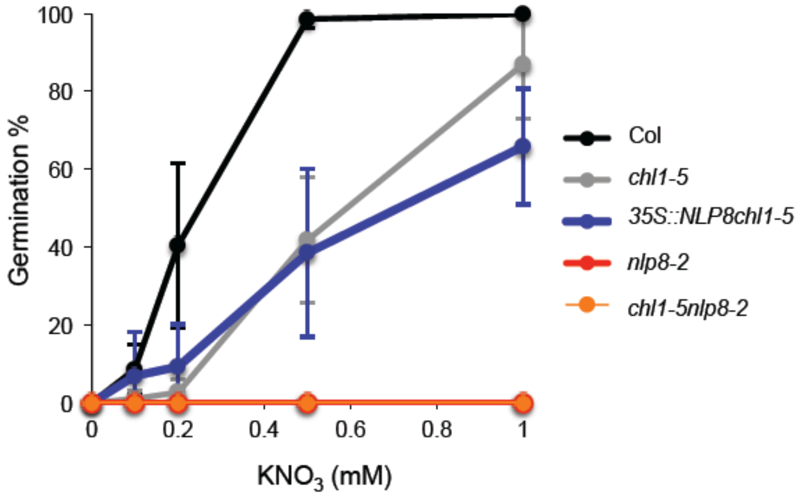

Supplementary Figure 10 | The effect of 35*S::NLP8* on the *chl1-5* mutation during germination.

Seeds of Col-0, *chl1-5*, *nlp8-2, chl1-5nlp8-2* mutants and *35S::NLP8 chl1-5* line were harvested from plants grown at 16°C, stored for 2 months. Germination test was performed using biological triplicates, and radicle protrusion through endosperm was counted 7 days after sowing. Percentage of germination under indicated KNO_3 concentrations is shown with SD. The numbers of biological replicates analyzed were as follows: Col-0, *n*=6; *chl1-5*, *n*=6; *35S::NLP8chl1-5*, *n*=6; *nlp8-2*, *n*=4; *chl1-5nlp8-2*, *n*=4.

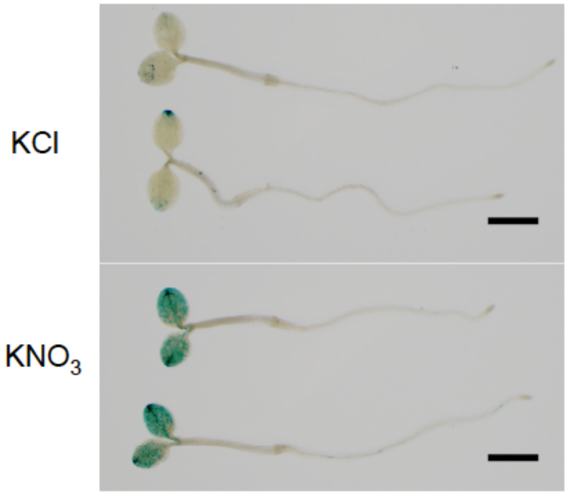

Supplementary Figure 11 | Nitrate-induced reporter expression.

GUS staining of transgenic lines harboring both effector (NLP8N-LexA_DB-YFP) and reporter (LexAOP-35Smini::GUS). Seven-day-old seedlings grown on KCl plate were transferred to liquid medium containing 3 mM KCl or KNO_3 for 4 hours and subjected to GUS staining. A bar indicates 5 mm.

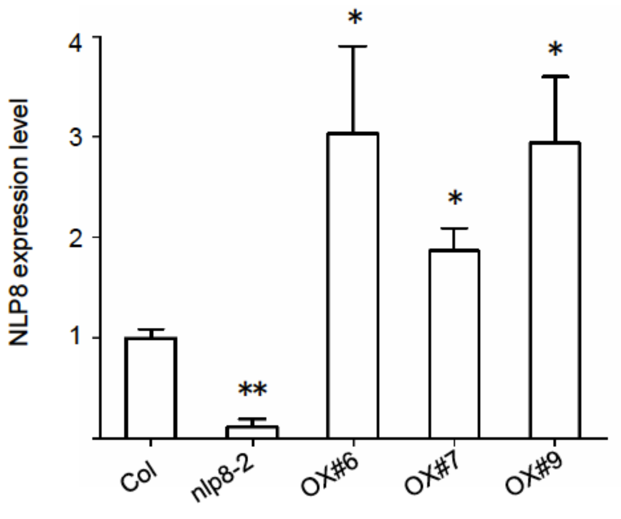

Supplementary Figure 12 | Expression of *NLP8* in 35S::NLP8-GFP lines.

Seeds of 35S::NLP8-GFP were harvested from plants grown at 16°C, stored for 2 months, imbibed for 6 hours in water with 1 mM KCl. qRT-PCR was performed with three biological replicates. Expression levels were normalized by that of At1g13320. Three lines showed similar results when analyzed. Data for line OX#6 is shown in the main figures. Averages of relative expression are shown with SD (n=3). * and ** indicate $P < 0.05$ and $P < 0.01$ (Student's t-test), respectively.

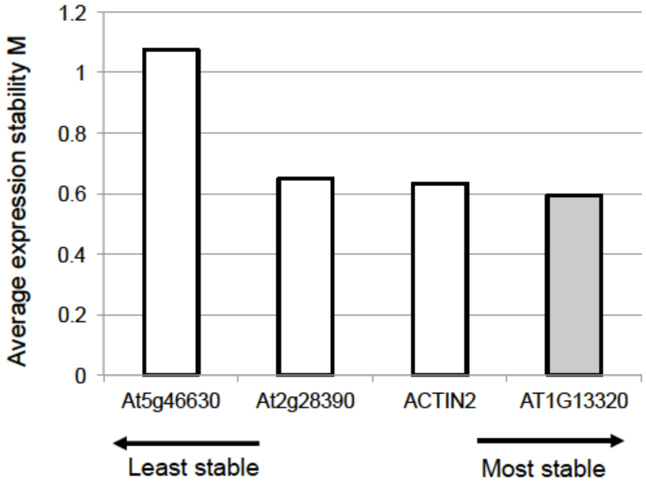

Supplementary Figure 13 | Expression stability of 4 reference genes.

qRT-PCR expression analysis was performed on 4 Arabidopsis reference genes published previously with cDNA from Col-0 and *nlp8-2* seeds imbibed in KCl or KNO_3 for 6 hours. Expression stability was analyzed by geNORM algorithm. Less M values indicate more stable as reference genes.
